# Insufficient epitope-specific T cell clones are responsible for impaired cellular immunity to inactivated SARS-CoV-2 vaccine in older adults

**DOI:** 10.1038/s43587-023-00379-0

**Published:** 2023-03-13

**Authors:** Chanchan Xiao, Zhiyao Ren, Bei Zhang, Lipeng Mao, Guodong Zhu, Lijuan Gao, Jun Su, Jiezhou Ye, Ze Long, Yue Zhu, Pengfei Chen, Xiangmeng Su, Tong Zhou, Yanhao Huang, Xiongfei Chen, Chaojun Xie, Jun Yuan, Yutian Hu, Jingshan Zheng, Zhigang Wang, Jianrong Lou, Xiang Yang, Zhiqiang Kuang, Hongyi Zhang, Pengcheng Wang, Xiaofeng Liang, Oscar Junhong Luo, Guobing Chen

**Affiliations:** 1 Department of Microbiology and Immunology; Institute of Geriatric Immunology; School of Medicine, Jinan University, Guangzhou, China; 2 Guangdong-Hong Kong-Macau Great Bay Area Geroscience Joint Laboratory, School of Medicine, Jinan University, Guangzhou, China; 3 Guangzhou Laboratory, Guangzhou, China; 4 Department of Systems Biomedical Sciences, School of Medicine, Jinan University, Guangzhou, China; 5 Guangzhou Geriatric Hospital, Guangzhou, China; 6 NHC Key Laboratory of Male Reproduction and Genetics, Guangzhou, China; 7 Department of Central Laboratory, Guangdong Provincial Reproductive Science Institute (Guangdong Provincial Fertility Hospital), Guangzhou, China; 8 Affiliated Huaqiao Hospital, Jinan University, Guangzhou, China; 9 Guangzhou Center for Disease Control and Prevention, Guangzhou, China; 10 Meng Yi Center Limited, Macau, China; 11 Shenzhen Kangtai Biological Products Co. Ltd, Shenzhen, China; 12 Leidebio Bioscience Co., Ltd., Guangzhou, China; 13 Department of Public Health and Preventive Medicine, School of Medicine, Jinan University, Guangzhou, China

**Keywords:** Infectious diseases, Infectious diseases, Ageing

## Abstract

Aging is a critical risk factor for severe acute respiratory syndrome coronavirus 2 (SARS-CoV-2) vaccine efficacy. The immune responses to inactivated vaccine for older adults, and the underlying mechanisms of potential differences to young adults, are still unclear. Here we show that neutralizing antibody production by older adults took a longer time to reach similar levels in young adults after inactivated SARS-CoV-2 vaccination. We screened SARS-CoV-2 variant strains for epitopes that stimulate specific CD8 T cell response, and older adults exhibited weaker CD8 T-cell-mediated responses to these epitopes. Comparison of lymphocyte transcriptomes from pre-vaccinated and post-vaccinated donors suggested that the older adults had impaired antigen processing and presentation capability. Single-cell sequencing revealed that older adults had less T cell clone expansion specific to SARS-CoV-2, likely due to inadequate immune receptor repertoire size and diversity. Our study provides mechanistic insights for weaker response to inactivated vaccine by older adults and suggests the need for further vaccination optimization for the old population.

## Abstract (web summary)

Xiao et al. report a weaker adaptive immune response in older adults after inactivated SARS-CoV-2 vaccination compared to young adult controls, identifying altered immune cell function and decreased antigen-specific receptor repertoire diversity as potential underlying mechanisms.

## Main

The ongoing Coronavirus Disease 2019 (COVID-19) pandemic, caused by severe acute respiratory syndrome coronavirus 2 (SARS-CoV-2), has resulted in nearly 628 million infections worldwide. The outcomes of viral infection vary broadly, with mild to moderate symptoms for most young individuals^1^. Age is the most important determinant of disease severity, with people over 65 years of age being at the greatest risk of requiring intensive care^2–4^. Old individuals showed the highest susceptibility to SARS-CoV-2, with higher hospitalization rates, severe illness rates and mortality^5,6^.

Immunosenescence is the gradual decline of the immune system brought on by aging, and age-associated changes in functionality and availability of T and B cells are thought to play an important role in decreased immune responses^7^. Eliciting neutralizing antibodies is one of the most common mechanisms for the current licensed COVID-19 vaccines^8–10^, and almost all neutralizing antibody responses, persistent antibody responses and affinity-matured memory B cells rely on the help of CD4 T cells^11^. In addition, studies also demonstrated the role of SARS-CoV-2-specific CD8 cytotoxic T lymphocytes and memory cells in convalescent COVID-19 patients^12–14^. It was reported that the weakened adaptive cellular immunity in old individuals appeared to be exacerbated during COVID-19, increasing the severity of the disease^15^. Another study also showed that coordination of SARS-CoV-2 antigen-specific immune responses, including antibody production and CD4 and CD8 T cell response, played a protective role in mild COVID-19 cases. However, this coordination was disrupted in individuals over 65 years of age and frequently failed to control the disease, indicating the connection between aging and impaired adaptive immune responses to the virus^16^. Therefore, there is an urgent need to assess specific T cells and neutralizing antibodies responding to vaccines in the old population.

Clinical trials with mRNA vaccines and adenovirus-vectored vaccines suggested lower antibody and T cell responses by old individuals^17–21^, but the underlying mechanisms were not thoroughly investigated. Inactivated virus vaccines have been administrated to adults between 18 years and 59 years of age in China since early 2021, and vaccination in individuals over 60 years of age started in July 2021. The inactivated virus vaccine CoronaVac was reported with adequate efficacy and induction of neutralizing antibodies in both young and old recipients^22,23^. However, comprehensive immune responses, including T cell responses, to the inactivated virus vaccines have not been systematically evaluated, especially for the old population.

To better understand the immune responses triggered by inactivated vaccines, we recruited young and old volunteers for a two-dose inoculation regimen with inactivated SARS-CoV-2 vaccines (CoronaVac and BBIBP-CorV) that have been widely administrated in China and multiple countries^24,25^, and a comprehensive comparison of vaccine-induced adaptive immune responses to SARS-CoV-2 was performed between the young and old volunteers, including specific anti-SARS-CoV-2 antibody productions and epitope-specific CD8 T cell responses. The results showed that the old individuals had worse CD8 T cell responses than antibody responses compared to the young individuals. The mechanisms were further revealed by the identification of altered immune cell gene expression and the markedly reduced antigen-specific CD8 T cell T cell receptor (TCR) repertoire in the old individuals. In the process, we also identified the dominant CD8 T cell epitopes containing the mutations from 13 circulating SARS-CoV-2 variants, including Alpha, Beta, Delta and Omicron variants, and compared the immune properties of the ancestral and mutant peptides.

## Results

### SARS-CoV-2 neutralizing antibody production comparison

**Fig. 1:**
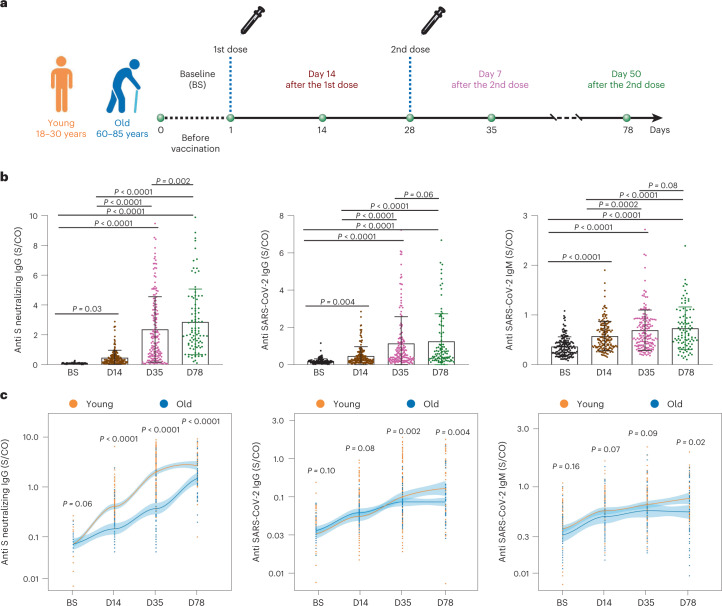
Study design and statistics of anti-SARS-CoV-2 antibodies in inactivated SARS-CoV-2 vaccine participants. **a**, Study design and sample collection timeline. Volunteer participants received two doses of the inactivated SARS-CoV-2 vaccine (CoronaVac or BBIBP-CorV), and blood samples were collected at indicated timepoints. **b**, SARS-CoV-2 neutralizing IgG, total IgG and IgM quantification by ELISA. The data are presented for all donors. BS, baseline, before vaccination; D14, 14 days after the first dose; D35, 35 days after the first dose, which is 7 days after the second dose; D78, 78 days after the first injection, which is 50 days after the second dose. **c**, Comparison of SARS-CoV-2 neutralizing antibody titer between young and old donors across the four timepoints. Young: 18–30-year-old healthy donor; Old: 60–85-year-old healthy donor. Significance was assessed by one-way ANOVA corrected for multiple comparisons using the least significant difference (LSD) method. Colored lines were fitted with cross-timepoint averages from each group with shading representing 95% confidence bands. Data are shown as mean ± s.d. *n* = 169 (121 young and 48 old) for BS, D14 and D35; *n* = 93 (45 young and 48 old) for D78. For **b** and **c**, each dot represents a single individual. *P* values were determined by one-way ordinary ANOVA.

**Extended Data Fig. 1:**
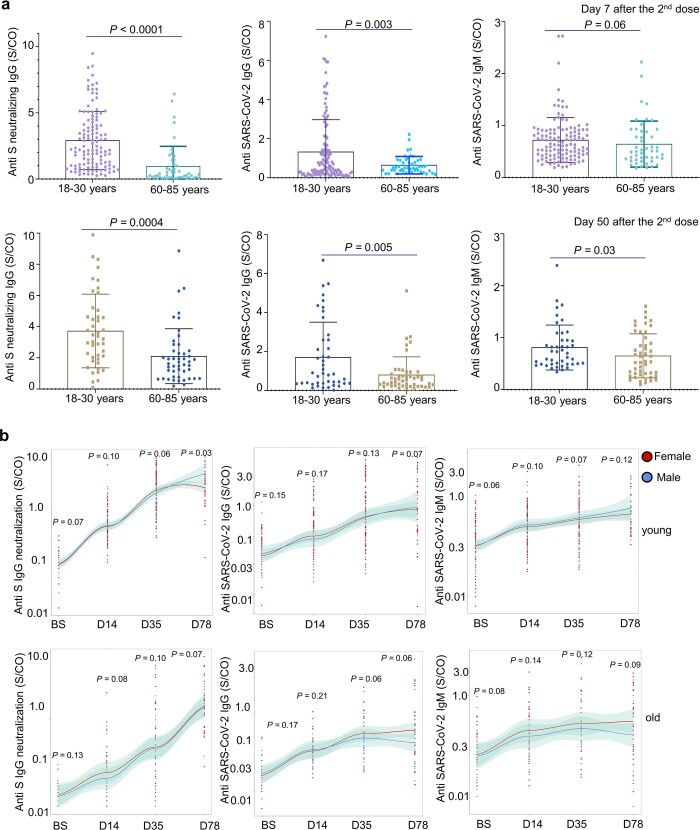
Statistics of anti-SARS-CoV-2 antibodies in inactivated SARS-CoV-2 vaccine recipients per age and gender group. (a) Comparison of SARS-CoV-2 neutralizing antibody titer between young and old group on Day 7 (top row) and Day 50 (bottom row) after receiving the second dose of inactivated SARS-CoV-2 vaccine. Young: 18-30 years old, n = 121 for Day 7 after the second dose, n = 44 for Day 50 after the second dose; Old: 60-85 years old, n = 48 for both Day 7 and Day 50 after the second dose. Each dot represents a single individual. Data are summarized as mean ± SD. P-values are determined by two-sided T-test. (b) SARS-CoV-2 neutralizing IgG, total IgG and IgM quantification by ELISA. The data are presented for the young (top panel) and old (bottom panel) participants separated by gender. The regression line is shown in red for female and blue for male, with shading representing the 95% confidence interval. Young: 18-30 years old, 81 females and 40 males; Old: 60-85 years old, 27 females and 21 males. D14: 14 days after the 1st injection; D35: 35 days after the 1st injection, which was 7 days after the 2nd dose; D78: 78 days after the 1st injection, which was 50 days after the 2nd dose. Each dot represents a single individual. Two-sided T-test was used for assessing the significance of difference between two gender groups at each time point.

To evaluate the adaptive immune response differences between young and old individuals after inactivated SARS-CoV-2 vaccine administration, we recruited a cohort of 121 healthy young (18–30 years old) and 48 old (60–85 years old) donors (Supplementary Table 1 and Methods). For each donor, the peripheral blood samples were collected for SARS-CoV-2 neutralizing antibody and T cell immune response examination at four timepoints: before vaccination (that is, baseline), on the 14th day after the first dose and on the 7th and 50th days after the second dose, respectively (Fig. 1a). As expected, the neutralizing antibody titers increased sequentially after vaccination for the entire cohort (Fig. 1b). However, on average, the old group exhibited lower neutralizing antibody titer increment and slower boosting rate compared to the young group (Fig. 1c and Extended Data Fig. 1a). Among the young adults, the males showed higher anti-S IgG production only at 50 days after the second dose (Extended Data Fig. 1b). The antibody production showed no significant difference between genders among the old individuals (Extended Data Fig. 1b). Notably, the inactivated virus vaccine was only able to induce sufficient neutralizing antibodies at the later timepoint (50 days after the second dose; Fig. 1c) but not at the earlier timepoint (7 days after the second dose) for the old group. The average neutralizing antibody level from the old group eventually reached the similar level of early time (7 days after the second dose) and 57% of later stage (50 days after the second dose) of the young group. Accordingly, antibody production after the two-dose vaccination in the old donors was slower and weaker, which suggests slower adaptive immune responses to inactivated SARS-CoV-2 vaccine by old individuals.

### Age group comparison of CD8 T-cell-mediated cytotoxic effect

**Fig. 2:**
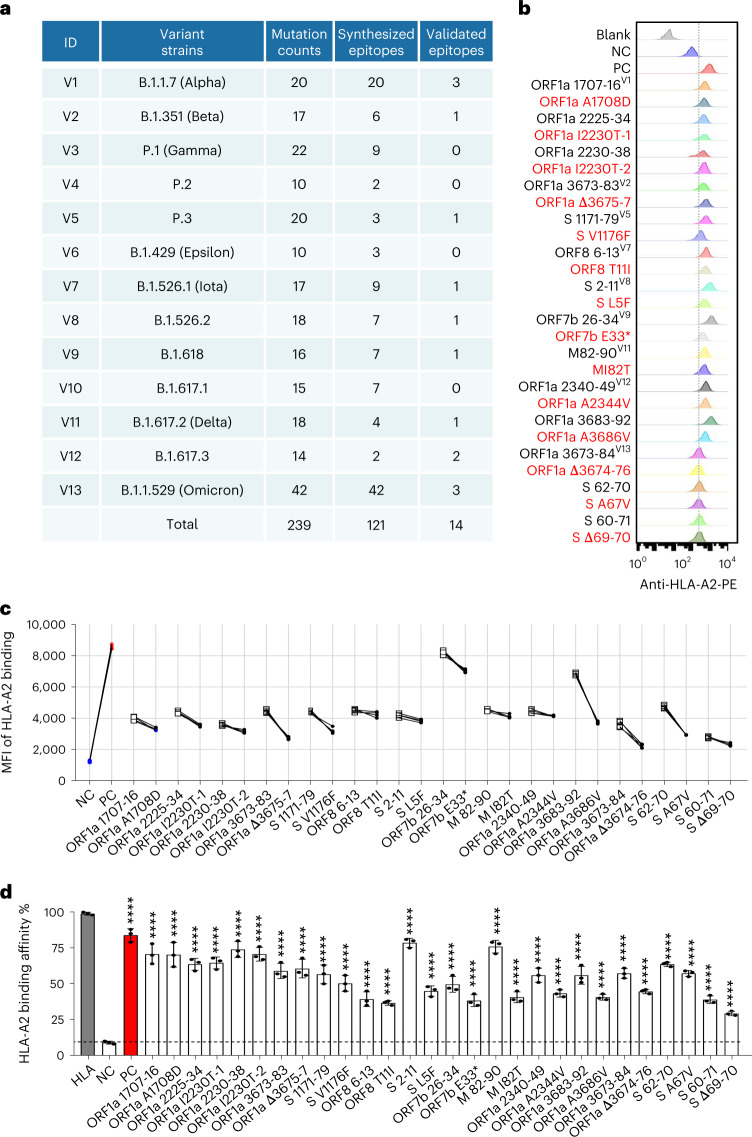
Identification of HLA-A2-restricted T cell epitopes from SARS-CoV-2 variants. **a**, Summary of mutation counts and synthesized and validated epitopes from 13 SARS-CoV-2 variant strains. **b**,**c**, Comparison of ancestral and mutant epitope binding affinity to HLA-A2 on T2 cells. Ancestral and mutant epitopes are listed in black and red, respectively, in **b**. Paired ancestral and mutant epitopes are listed adjacently. Numeric superscripts in **b** correspond to ID numbers in **a**. Blank, no peptides; NC, negative control, EBV virus peptide IVTDFSVIK; PC, positive control, influenza A M1 peptide GILGFVFTL. The same applies throughout the paper. **d**, Evaluation of ancestral and mutant SARS-CoV-2 epitope binding to HLA-A2 by ELISA assay. Data are shown as mean ± s.d., *n* = 3 independent experiments for each tested epitope. ****P < 0.0001 (two-sided *t*-test, comparing to NC). Threshold for peptide MHC (pMHC) formation positivity was set as above the average OD value of the negative control. HLA: control UV-sensitive peptide without UV irradiation.

**Fig. 3:**
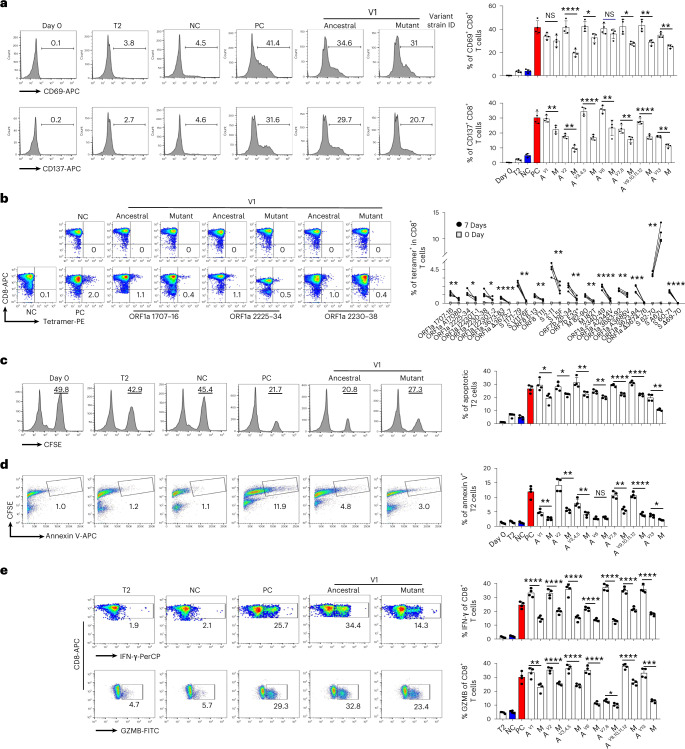
Activation of CD8 T cells by epitopes from SARS-CoV-2. **a**, Exemplary flow cytometry result (left) and overall summary (right) of CD8 T cell activation marker CD69 and CD137 expression after co-cultivation with T2 cells loaded with a distinct set of peptides (*n* = 4). CD69 and CD137 expression was detected by FACS 16 hours after co-cultivation. Paired ancestral and mutant epitopes are placed adjacently. A, ancestral; M, mutant. Variant strain IDs indicate mixed ancestral or mutant epitopes from the corresponding variant strain in Fig. 2a. The same applies throughout the paper. **b**, Left: representative FACS plots of specific CD8 T cells recognized by tetramers containing SARS-CoV-2 epitope. Top row, day 0; bottom row, day 7. CD8 T cells from healthy donors were co-cultivated with T2 cells loaded with epitopes for activation. Right: epitope-specific CD8 T cell quantification (*n* = 4) before (day 0) and after 7-day stimulation by distinct SARS-CoV-2 epitopes. *n* = 3 for S 269–277 YLQ and S P272L. **c**, Epitope-specific CD8 T-cell-mediated cytotoxicity evaluation after 7 days of cell culturing. Left: exemplary flow cytometry results. The CFSE+ T2 cells were counted as survived target cells and are presented as percentage. Right: corresponding summary statistics for all tested epitopes; percentage of apoptotic cells was calculated by 50% minus the percentage of survived cells (*n* = 4). **d**, Left: exemplary FACS result showing the percentage of CFSE+ Annexin V+ T2 cells presenting distinct antigens after 7 days of culturing with CD8 T cells, as indicator of epitope-stimulated T-cell-mediated T2 apoptosis. Right: corresponding summary statistics for all tested epitopes (*n* = 4). **e**, Left: expression of IFN-γ (top row) and GZMB (bottom row) by CD8 T cells after epitope stimulation for 7 days (*n* = 4). Values in each panel indicate the percentage of IFN-γ^+^CD8^+^ or GZMB^+^CD8^+^ T cells, respectively. Right: corresponding summary statistics for all tested epitopes (*n* = 4). Data shown are mean ± s.d. Each dot represents a single experiment. Statistical significance was determined by one-sided *t*-test or one-way ANOVA. ****P < 0.0001, ***P < 0.001, **P < 0.01, *P < 0.05 and NS, not statistically significant (*P* ≥ 0.05).

We then focused on how antigen-specific CD8 T cell immune response changed after vaccination by examining how CD8 T cells of the vaccinated individuals reacted to the SARS-CoV-2 antigenic epitopes. To do so, we first identified all possible SARS-CoV-2-specific epitopes for CD8 T cells, including the ones from the original ancestral and 13 emerging variant strains. In our previous study, we identified ten HLA-A2-restricted epitopes from spike, envelope and membrane protein of the ancestral strain (Wuhan-Hu-1) of SARS-CoV-2 (refs. ^26,27^). In the present study, we globally predicted all the potential HLA-A2-restricted epitopes from the 13 variant strains (Fig. 2a), including the Alpha, Beta, Delta and Omicron strains that caused major pandemics globally. We focused on the HLA-A2 major histocompatibility complex class I (MHC-I) molecule as it is the most common among the Chinese population^28,29^. In total, 121 pairs of predicted epitopes from the variant strains, together with the corresponding epitopes from the ancestral strain, were synthesized for MHC-I binding and T cell activation capability screening. The results showed that 103, 13, 1 and 4 of the mutant epitopes, relative to the ancestral, exhibited none, impaired, unchanged or increased MHC-I binding affinity, respectively (Figs. 2a-d and 3a-e, Supplementary Table 2 and Methods). Based on the criterion of the proportion of ancestral peptide-activated T cells greater than 1%, we then focused on the 14 pairs of epitopes with the mutant causing impaired MHC-I binding, which were located in ORF1a, spike (S), ORF8, ORF7b and membrane (M) proteins, respectively. The T2 binding assay (Methods) showed decreased MHC-I binding capability by the variant-mutated epitopes compared to the corresponding ancestral peptides (Fig. 2b,c and Supplementary Table 2). However, these epitopes could still be constructed as peptide–MHC monomers and further tetramers (Fig. 2d and Supplementary Table 2).

**Extended Data Fig. 2:**
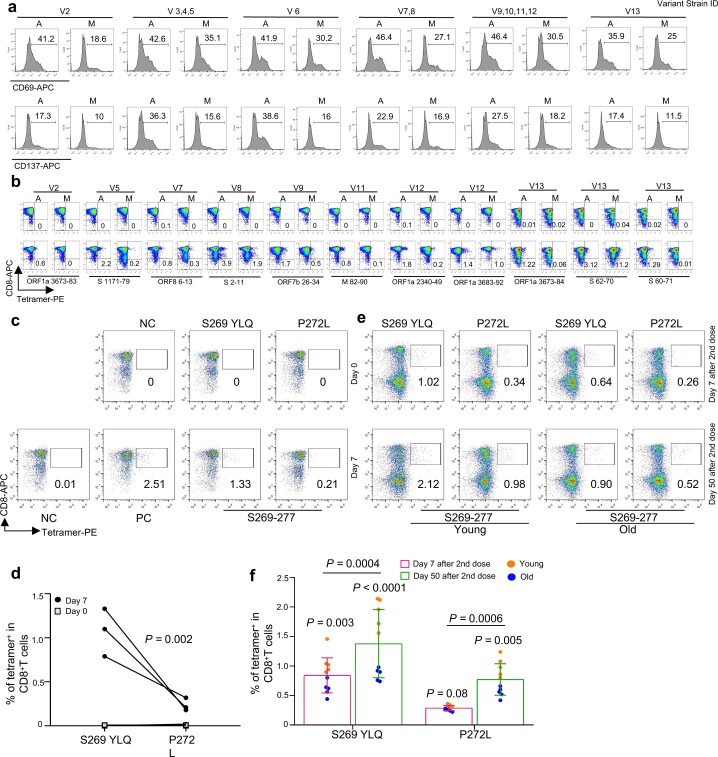
Identification of HLA-A2-restricted T cell epitopes and activation of CD8 T cells by epitopes from SARS-CoV-2. (a) Exemplary flow cytometry result of CD8 T cell activation marker CD69 and CD137 expression after cocultivation with T2 cells loaded with distinct set of peptides (n = 4 per experiment). Variant strain IDs indicate mixed ancestral or mutant epitopes from the corresponding variant strain in Fig. 2a that were used in each experiment. CD69 and CD137 expression was detected by flow cytometry 16 hours after cocultivation. A: ancestral; M: mutant. Paired ancestral and mutant epitopes are placed adjacently. (b) Representative FACS plots of specific CD8 T cells recognized by tetramers containing SARS-CoV-2 epitope peptides. CD8 T cells from healthy donors were co-cultivated with T2 cells loaded with various peptides for activation for 16 hours. The cells were stained with corresponding tetramer containing ancestral or mutated epitope, and compared before (Day 0, top row) and after (Day 7, bottom row) stimulation. (c) Representative FACS plots of specific CD8 T cells recognized by tetramers containing SARS-CoV-2 epitope peptides. CD8 T cells from healthy donors were co-cultivated with T2 cells loaded with tetramers prepared using SARS-CoV-2 epitope S269-277. The cells were stained with corresponding tetramer containing ancestral or mutated epitope, and compared before (Day 0; top row) and after (Day 7; bottom row) stimulation. (d) Epitope-specific CD8 T cell quantification (n = 3) before (day 0) and after 7 days of stimulation by distinct pair of ancestral and mutant SARS-CoV-2 epitopes. P-values are determined by two-sided T-test. (e) Representative data for detection of epitope-specific CD8 T cells in the HLA-A2^+^ healthy donors after second doses (Day 7 and Day 50) of inactivated SARS-CoV-2 vaccine with tetramers prepared using SARS-CoV-2 epitope S269-277 for. Cells were sitmulated for 16 hours before staining. (f) Comparison of epitope-specific CD8 T cells between HLA-A2^+^ healthy young (n = 5) and old (n = 5) donors, 7 (top row) and 50 (bottom row) days after second doses of inactivated SARS-CoV-2 vaccine. Specific CD8 T cells were stained with tetramers prepared using ancestral (S269-277) and mutant (P272L) SARS-CoV-2 epitopes individually. Data are summarized as mean ± SD. Paired ancestral and mutant epitopes are listed adjacently on the x-axis. P-values are determined by two-sided T-test.

**Extended Data Fig. 3:**
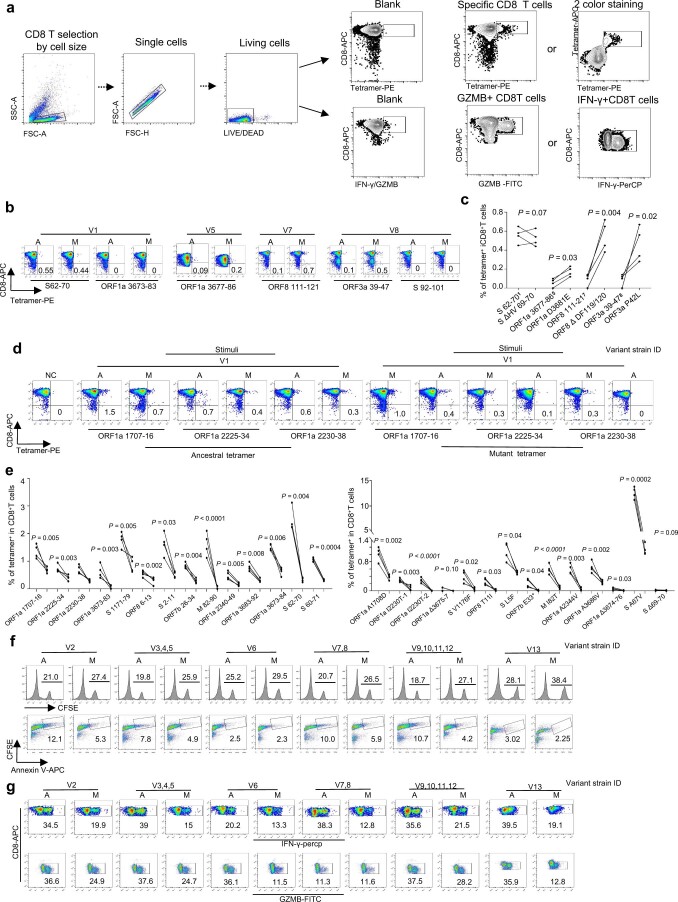
Immune response alteration by SARS-CoV-2 mutant epitopes. (a) Flow cytometry gating strategy for SRAS-CoV-2 epitope specific CD8 T, GZMB+ CD8 T and IFNγ+ CD8 T cells, respectively. (b) Representative FACS results for CD8 T cell selection by tetramers containing SARS-CoV-2 epitope peptides. CD8 T cells from healthy donors were co-cultivated with T2 cells loaded with various peptides for specific CD8 T cell recognition. The cells were stained with corresponding tetramer containing ancestral or mutated epitope after 7 days. (c) Statistics of ancestral or mutant SARS-CoV-2 epitope specific CD8 T cells (n = 4). The pairs of ancestral and mutant epitopes shown here produced unchanged or increased CD8 T cell binding efficiency by mutated epitope comparing to ancestral. P-values are determined by two-sided T-test. (d) Cross-detection of epitope specific CD8 T cells with tetramers based on ancestral and corresponding mutant peptides. Left: ancestral or mutant epitopes stimulated CD8 T cells stained with ancestral peptide-based tetramer; Right: mutant or ancestral epitopes stimulated CD8 T cells stained with mutant peptide-based tetramer. (e) Statistics of cross-detection of epitope specific CD8 T cells (n = 4). Left: ancestral or mutant epitopes stimulated CD8 T cells stained with ancestral peptide-based tetramer; Right: mutant or ancestral epitopes stimulated CD8 T cells stained with mutant peptide-based tetramer. The P-values are calculated by paired t-test. NC: negative control, EBV virus peptide IVTDFSVIK. (f) Specific CD8 T cell mediated cytotoxicity evaluation after stimulation by ancestral and mutant epitopes from additional variant strains for 7 days. The remained CFSE labeled T2 cells were counted and presented as survived target cells (top row). The values in the flow cytometry chart indicate the percentage of surviving T2 cells. The proportion of CFSE+ Annexin V+ T2 cells presenting distinct SARS-CoV-2 antigens after 7 days culturing with CD8 T cells, as indicator for epitope stimulated T cell mediated T2 apoptosis (bottom row). (g) Detection of IFN-γ+ (top row) and Granzyme B+ (bottom row) CD8 T cells after stimulation by ancestral and mutant epitopes from additional variant strains for 7 days. Values in each panel indicate the percentage of IFN-γ+ CD8 + and Granzyme B+ CD8+ T cells, respectively. Variant strain ID numbers corresponds to SARS-CoV-2 variant strain IDs listed in Fig. 2a.

In the T cell activation assay using CD8 T cells from healthy HLA-A2^+^ donors, most of the T cells exhibited decreased activation upon stimulation by the mutated epitopes, as indicated by CD69 and CD137 fluorescence-activated cell sorting (FACS) signals, comparing to the ancestral peptides (Fig. 3a and Extended Data Fig. 2a). The tetramer staining showed significant reduction of epitope-specific CD8 T cells in the mutated compared to the corresponding ancestral (Fig. 3b, Extended Data Figs. 2b–d and 3a–c and Supplementary Table 2). We also tested the previously reported HLA-A2-restricted SARS-CoV-2 epitope S 269–277 YLQ and its corresponding P272L mutant (Extended Data Fig. 2c,d)^30–33^. Furthermore, the CD8 T cells stimulated with ancestral and mutant epitopes could not be cross-detected by the mutant and ancestral epitope-based tetramers, respectively (Extended Data Fig. 3d,e), which suggested that the establishment of new immune responses was required for the mutated epitopes from the given SARS-CoV-2 variants. The cytotoxicity assay also showed impaired cytotoxic capability by CD8 T cells stimulated by the mutant epitopes, with decreased killing of target cells (Fig. 3c,d and Extended Data Fig. 3f). The induced IFN-γ and Granzyme B (GZMB) levels were also reduced in the mutant group comparing to the ancestral (Fig. 3e and Extended Data Fig. 3g). Taken together, we identified 14 pairs of ancestral and mutant SARS-CoV-2 epitopes (HLA-A2-restricted) inducing CD8 T cell immune response, with the mutant ones from the variant strains causing impaired immune function.

**Fig. 4:**
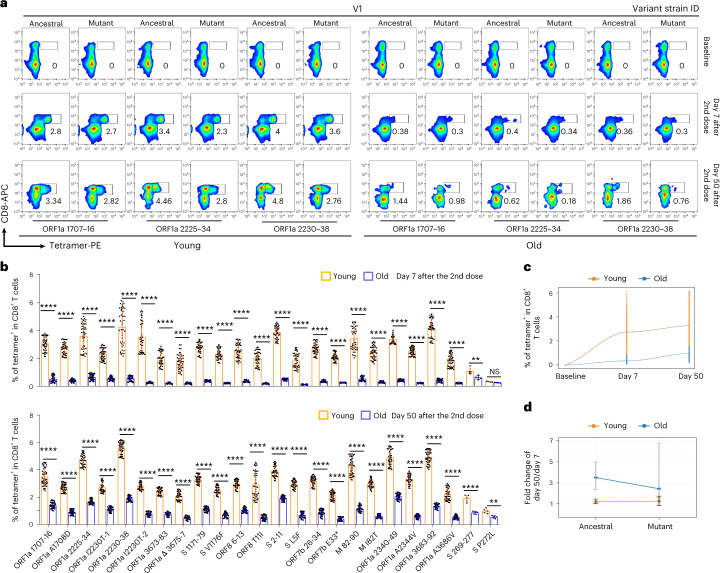
Comparison of SARS-CoV-2 epitope-specific CD8 T cells between young and old vaccine recipients. **a**, Representative data for in vitro detection of epitope-specific CD8 T cells in the HLA-A2^+^ healthy donors before and after second doses (7 days and 50 days) of inactivated SARS-CoV-2 vaccine with tetramers prepared using SARS-CoV-2 epitopes. Variant strain IDs are the same as listed in Fig. 2a. Cells were stimulated for 16 hours before tetramer staining. The flow cytometry gating strategy is shown in Extended Data Fig. 4a. **b**, Comparison of epitope-specific CD8 T cells between HLA-A2^+^ healthy young and old donors, 7 days (top row) and 50 days (bottom row) after second doses of inactivated SARS-CoV-2 vaccine. Specific CD8 T cells were stained with tetramers prepared using ancestral and mutant SARS-CoV-2 epitope individually after 16-hour stimulation. Paired ancestral and mutant epitopes are listed adjacently on the *x* axis. Data are shown as mean ± s.d. *n* = 5 for S 269–277 and S P272L; otherwise, *n* = 45 in all the other tests for both the young and old groups. ****P < 0.0001, ***P < 0.001, **P < 0.01, *P < 0.05 and NS, not statistically significant (*P* ≥ 0.05), two-sided *t*-test. **c**, Overall statistics and comparison of SARS-CoV-2 epitope-specific CD8 T cells on the 7th and 50th days after the second dose in young and old recipients. **d**, Summary statistics of detection fold change of CD8 T cells specific to SARS-CoV-2 epitopes between 7 days and 50 days after the second dose. Data shown are mean ± s.d., *n* = 45 for both the young and old groups.

**Extended Data Fig. 4:**
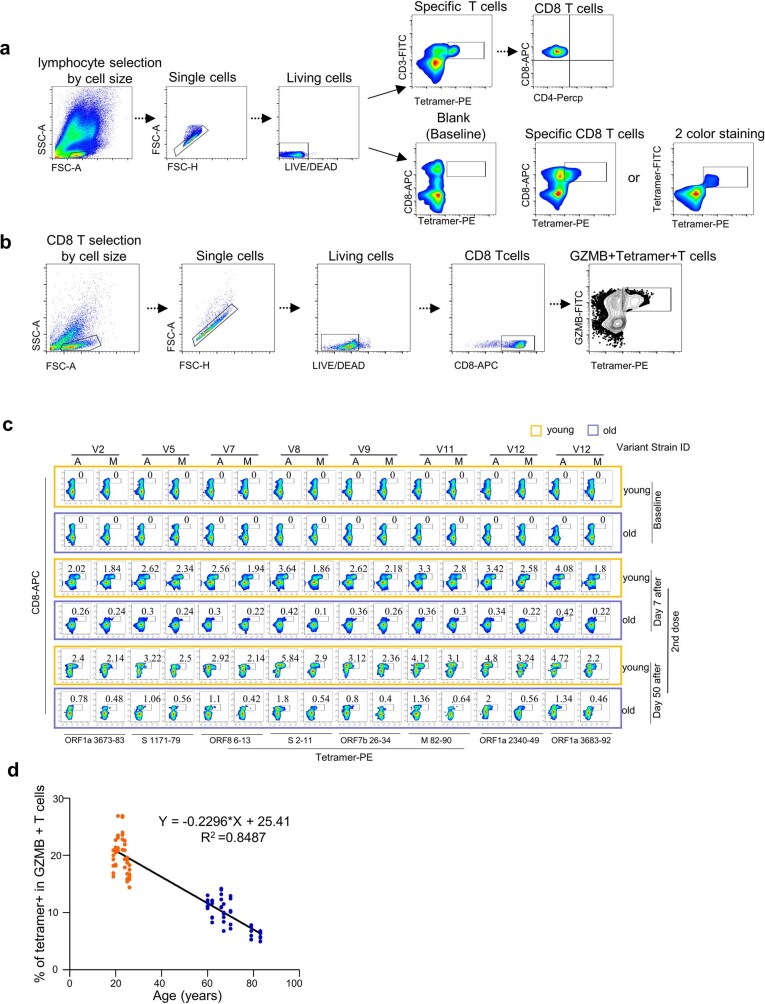
Flow cytometry gating strategies for SARS-CoV-2 epitope specific CD8 T cells and GZMB+ CD8 T cells, and detection and characterization of specific CD8 T cells and GZMB+ CD8 T cells in young and old vaccine recipients. (a) Flow cytometry gating strategy for SARS-CoV-2 epitope specific CD8 T cells. (b) Flow cytometry gating strategy for SARS-CoV-2 epitope specific and GZMB+ CD8 T cells. (c) Representative data for detection of epitope specific CD8 T cells in the HLA-A2^+^ healthy donors before and after second doses (Day 7 and Day 50) of inactivated SARS-CoV-2 vaccine with tetramers prepared using SARS-CoV-2 epitopes. Variant strain IDs are listed in Fig. 2a. A: ancestral; M: mutated. The flow cytometry gating strategy is shown in (a). (d) Trends in tetramer+GZMB+ T cells production with age in young and old groups after the second dose vaccination. n = 6 individuals for each group, and each individual with 7 experiments corresponding to 7 distinct epitopes as listed in Fig. 5c.

**Extended Data Fig. 5:**
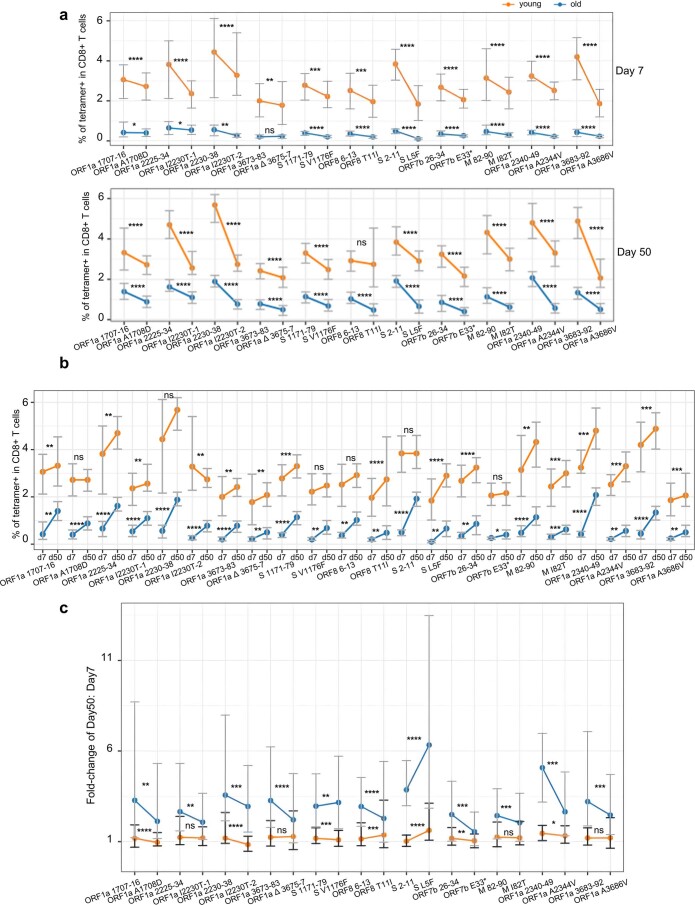
Comparisons of ancestral and mutant SARS-CoV-2 epitope specific CD8 T cells between young and old vaccine recipients. (a) Comparison of specific CD8 T cells between ancestral and mutant SARS-CoV-2 epitopes in HLA-A2^+^ donors, 7 (top) and 50 (bottom) days after the second dose. Specific CD8 T cells were stained with tetramers prepared using ancestral and mutant SARS-CoV-2 epitope individually. Paired ancestral and mutant epitopes are listed adjacently on x-axis. Data shown are mean ± SD. n = 45 for both young and old group. ****: *p* < 0.0001, ***: *p* < 0.001, **: *p* < 0.01, *: *p* < 0.05, ns: not statistically significant (p ≥ 0.05); P-values are determined by two-sided T-test. (b) Comparison of SARS-CoV-2 epitopes specific CD8 T cells on the 7th and 50th day after the second vaccination in young and old recipients. Paired ancestral and mutant epitopes are listed adjacently on x-axis. Data shown are mean ± SD. n = 45 for both young and old group. ****: *p* < 0.0001, ***: *p* < 0.001, **: *p* < 0.01, *: *p* < 0.05, ns: not statistically significant (p ≥ 0.05); P-values are determined by two-sided T-test. (c) Detection fold-change of CD8 T cells specific to SARS-CoV-2 epitopes between 50 and 7 days after the second dose. Paired ancestral and mutant epitopes are listed adjacently on x-axis. Data shown are mean ± SD. n = 45 for both young and old group. ****: *p* < 0.0001, ***: *p* < 0.001, **: *p* < 0.01, *: *p* < 0.05, ns: not statistically significant (*p* ≥ 0.05); P-values are determined by two-sided T-test.

We then constructed epitope-based tetramers to access the production of SARS-CoV-2 antigen-specific CD8 T cells in the young and old individuals after inactivated SARS-CoV-2 vaccine administration. Based on the tetramer staining, SARS-CoV-2 epitope-specific CD8 T cells were detected in all HLA-A2^+^ donors after vaccination (Fig. 4a,b and Extended Data Figs. 2e,f and 4a–c). However, the epitope-specific CD8 T cells were significantly less in the old donors compared to the young donors (0.35% ± 0.17% in old versus 2.76% ± 0.96% in young on day 7 after the second dose; 1.02% ± 0.52% in old versus 3.31% ± 1.41% in young on day 50 after the second dose) (Fig. 4a–c and Extended Data Fig. 5a,b). Furthermore, antigen mutation caused by variant strains led to decreased amount of SARS-CoV-2-specific CD8 T cells in both the young group (ancestral: 3.23% ± 0.95% versus mutant: 2.29% ± 1.13% on day 7 and ancestral: 3.96% ± 1.52% versus mutant: 2.65% ± 1.08% on day 50 after the second dose) and the old group (ancestral: 0.44% ± 0.17% versus mutant: 0.26% ± 0.13% on day 7 and ancestral: 1.38% ± 0.46% versus mutant: 0.66% ± 0.24% on day 50 after the second dose), indicating potential immune escape of the variant strains (Fig. 4b).

Across the samples collected through the vaccination course, the amount of CD8 T cells specific to most epitopes increased from day 7 to day 50 after the second dose in both the young donors (from 2.76% to 3.31% on average, 1.19-fold increase) and old donors (from 0.35% to 1.02% on average, 2.91-fold increase), and the increment in the old donors was stronger; however, the eventual epitope-specific CD8 T cells in old donors was only, on average, 12.68% and 30.81% of the young group on day 7 and day 50 after the second dose, respectively (Fig. 4c and Extended Data Fig. 5a). In addition, the increment of specific CD8 T cells between day 7 and day 50 after the second dose in the old group was greater than in the young group for both ancestral and mutant epitopes (Fig. 4d and Extended Data Fig. 5b,c). These results indicated that the CD8 T cell response in old donors required longer time to be eventually established, and the response was more disturbed by mutations from variant strains.

**Fig. 5:**
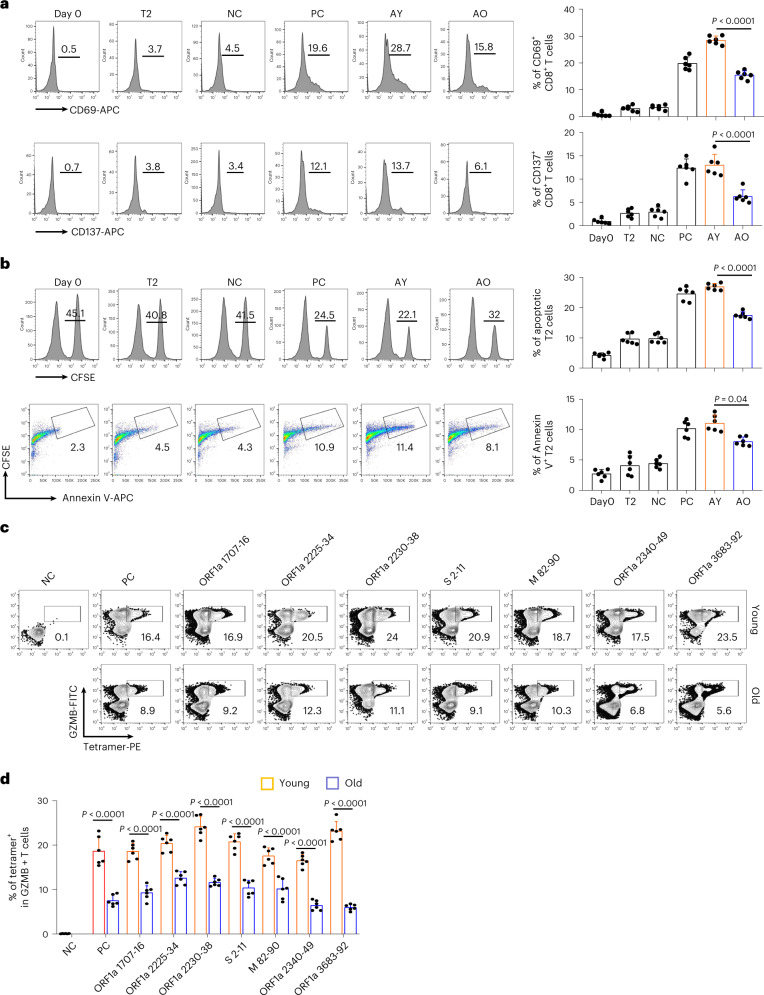
Comparison and characterization of cytotoxic effects of SARS-CoV-2 epitope-specific CD8 T cells between young and old vaccine recipients. **a**–**d**, Characterization of epitope-specific CD8 T cells after vaccination. CD8 T cells isolated from vaccinated donors after the second dose (day 50) were co-cultivated with T2 cells loaded with SARS-CoV-2 epitopes at a 1:1 ratio and analyzed for the expression of CD69 and CD137 after 16 hours (**a**), for target cell cytotoxicity (**b**) and for GZMB production after 7 days (**c** and **d**). For the cell cytotoxicity assay, T2 cells were labeled with CFSE and loaded with SARS-CoV-2 epitopes (ORF1a 1707–1716, ORF1a 2225–2234, ORF1a 2230–2238, S 2–11, M 82–90, ORF1a 2340–2349 and ORF1a 3683–3692) as target cells. Target cell cytotoxicity was assessed by the proportion of killed T2 cells and the apoptotic T2 cells. Day 0, control before stimulation; T2, T2 control cells without any peptide; NC, negative control, T2 cells loaded with EBV virus peptide IVTDFSVIK; PC, positive control, T2 cells loaded with influenza A M1 peptide GILGFVFTL; AY, co-cultivation with CD8 T cells from young donors; AO, co-cultivation with CD8 T cells from old donors. Data are summarized as mean ± s.d. *n* = 6 for each group. Statistical significance was determined by one-sided *t*-test or one-way ANOVA. The flow cytometry gating strategy is shown in Extended Data Fig. 4b.

Furthermore, we selected epitopes with specific CD8 T cell above 3% and 0.4% on the 7th day after the second dose in young and old donors, respectively, for cytotoxic effects comparison. The results indicated that the vaccination-stimulated CD8 T cells from the old group exhibited lower expression levels of CD69 and CD137 (Fig. 5a), impaired cytotoxic effect on target cells (Fig. 5b) and decreased GZMB (Fig. 5c,d and Extended Data Fig.4d) production compared to the young group. All these results suggested that the inactivated SARS-CoV-2 vaccine could stimulate to produce functional antigen-specific CD8 T cells in the old individuals, but the degree was significantly less than in young individuals.

### Comparison of lymphocyte transcriptomes between young and old individuals

**Fig. 6:**
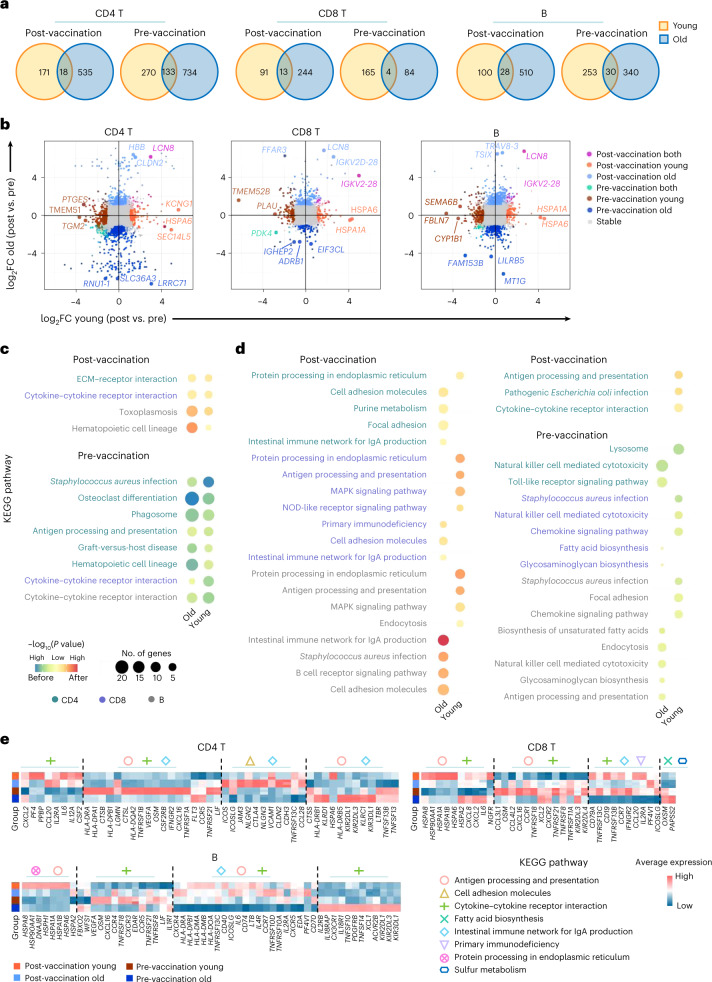
Transcriptomic comparison of CD4 T, CD8 T and B cells between young and old donors before and after vaccination. **a**, Venn diagrams of the significantly upregulated genes before and after vaccination in the young and old donors, from CD4 T, CD8 T and B cells, respectively. **b**, Scatter plot visualization of gene expression fold changes between pre-vaccination and post-vaccination in CD4 T, CD8 T and B cells from young and old donors. The top three DEGs between pre-/post-vaccination identified from young/old donors or both are labeled. See complete DEG list in Supplementary Table 3. **c**, KEGG pathway enrichment for vaccination-condition-specific genes identified from young and old donors. Yellow–blue color scale corresponds to the enrichment significance of the pre-vaccination upregulated genes; yellow–red color scale corresponds to the opposite. Dot size is proportional to the number of genes annotated to the corresponding pathway. *P* values are calculated by Fisherʼs exact test. **d**, KEGG pathway enrichment for vaccination-condition-specific genes identified from young or old donors. Color scale and dot size scale are the same as **c**. **e**, Heat map visualization of the expression levels of DEGs annotated to selected KEGG pathways. Heat map color reflects the average normalized expression levels across samples (*n* = 3) within each group. ECM, extracellular matrix; FC, fold change.

**Extended Data Fig. 6:**
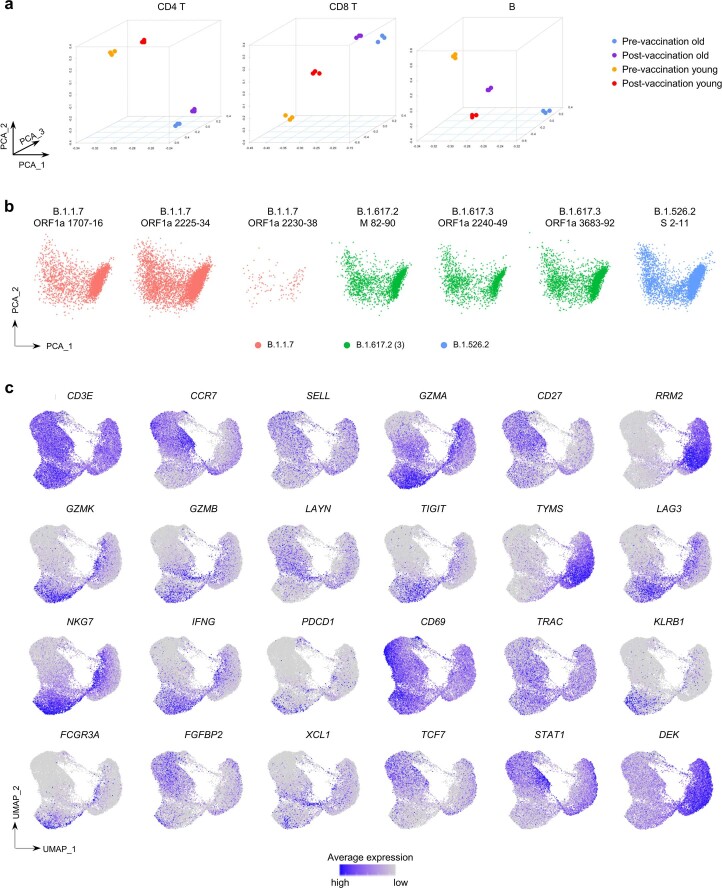
PCA analyses of bulk cell RNA-Seq and scRNA-Seq data, and visualization of selected marker gene expression. (a) PCA visualization of transcriptome profile from bulk cell RNA-Seq for CD4 T, CD8 T and B cells between young and old donors before and after vaccination. (b) PCA visualization of single-cell transcriptome profile for each sample of CD8 T cells specific to 7 distinct SARS-CoV-2 epitopes. The PCA plots showed no obvious batch effect among the analyzed scRNA-Seq datasets. (c) UMAP visualization of scRNA-Seq data with selected marker gene expression projection.

To comprehensively characterize the adaptive immune responses and understand the potential mechanisms behind the differences in antibody production and cytotoxic effects between the young and old individuals after vaccination, RNA sequencing (RNA-seq) transcriptome analyses were performed for CD4 T, CD8 T and B cells with triplicate samples collected before and after vaccination (7 days after the second dose; Methods). Principal component analysis (PCA) showed that samples from each group and condition were clustered and separated from the others (Extended Data Fig. 6a). The significant differentially expressed genes (DEGs) between pre-vaccination and post-vaccination were first detected for the young and old groups, respectively. Then, the common upregulated genes among young and old groups from each vaccination condition and cell type combination were identified (Fig. 6a,b). Only a small number of vaccination-condition-specific upregulated genes (from 1.6% to 11.7%) were shared among the young and old individuals in each screened cell type—that is, most vaccination-condition-specific genes were age group specific (Fig. 6a,b and Supplementary Table 3). Next, we sought to understand the functional implication of the condition-specific upregulated genes by KEGG pathway enrichment analysis (Methods). The results revealed that only a few functional pathways were of significant enrichment for the common DEGs before and after vaccine injection (Fig. 6c and Supplementary Table 4). This is most likely because the number of age-group-shared DEGs per vaccination condition was small. In contrast, many more functional pathway enrichments were identified for age-group-specific genes both before and after vaccination (Fig. 6d and Supplementary Table 4).

Functional enrichment analysis of the transcriptome data indicated that the lymphocytes from young and old donors reacted differently after encountering inactivated SARS-CoV-2 vaccine. Under resting condition before vaccination, immune cells from the old individuals were enriched with upregulated genes annotated to innate immune cell function (natural killer cell-mediated cytotoxicity, toll-like receptor signaling pathway and endocytosis), metabolism alteration (fatty acid and glycosaminoglycan biosynthesis) and change of antigen presentation on antigen-presenting B cells, suggesting potential ‘inflamm-ageing’ in the old individuals^34^ (Fig. 6d). After vaccination, immune cells from the young group had strong upregulation of genes involved in immune response, such as protein processing for immune response, antigen processing and presentation and MAPK signaling pathway. However, none of these enrichments was found in the old group, but pathways related to cell migration (cell adhesion molecules and focal adhesion), immunodeficiency and IgA production were found, suggesting the potential disability of processing the viral proteins from the vaccine and presenting them as peptide antigens by the lymphocytes of the old donors (Fig. 6d,e and Supplementary Table 4).

### SARS-CoV-2 epitope-specific TCR repertoire

**Fig. 7:**
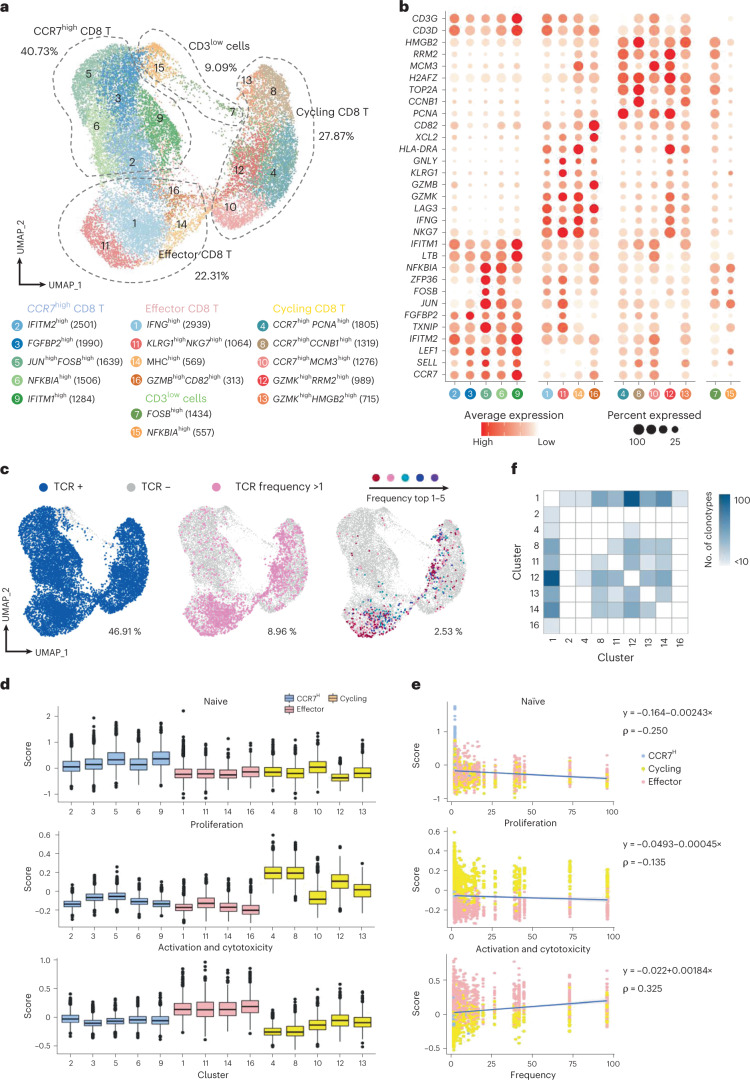
Single-cell transcriptome and TCR landscape of CD8 T cells specific to the top seven SARS-CoV-2 ancestral epitopes by CD8 cell activation capacity. **a**, Uniform manifold approximation and projection (UMAP) visualization of the SARS-CoV-2 epitope-specific CD8 T cells, by single-cell transcriptome profiles. Clusters are named based on the cluster-specific marker genes. The numbers in parentheses indicate the number of cells in each cluster. The SARS-CoV-2 epitopes are listed in Fig. 5c. **b**, Dot plot of marker genes for each CD8 T cell subtypes. Color scale shows the average normalized expression of marker genes in each subtype, and dot size indicates the percentage of cells within each cell cluster expressing the marker gene. **c**, Same UMAP visualization as **a** but with total TCR sequence detection information (left), TCR clonotype expansion (clonotype frequency >1) information (middle) and the top five most frequent TCR clonotype information for the seven ancestral epitopes projection. **d**, Single-cell transcriptome-derived CD8 T cell naivety, proliferation and activation and cytotoxicity score comparison between cell clusters. Number of cells (*n*) in each tested cluster is shown in **a**. Gene panels used for naivety, proliferation and activation and cytotoxicity score calculation are listed in Supplementary Table 6. **e**, Scatter plot visualization of CD8 T cell TCR clonotype frequency versus cell naivety, proliferation and activation and cytotoxicity score, respectively. Each dot represents a CD8 T cell, with color corresponding to its annotated subtype. Blue lines are fitted by linear model, with gray error bands indicating the 95% confidence intervals. Spearman correlations (ρ) are also shown. **f**, Heat map visualization of numbers of TCR clonotypes shared by two CD8 T cell clusters. Only cell clusters with shared clonotypes >10 with at least one other cluster are shown. For box plots, the outlines of the boxes represent the first and third quartiles; the line inside each box represents the median; and boundaries of the whiskers are found within the 1.5× interquartile range value.

**Extended Data Fig. 7:**
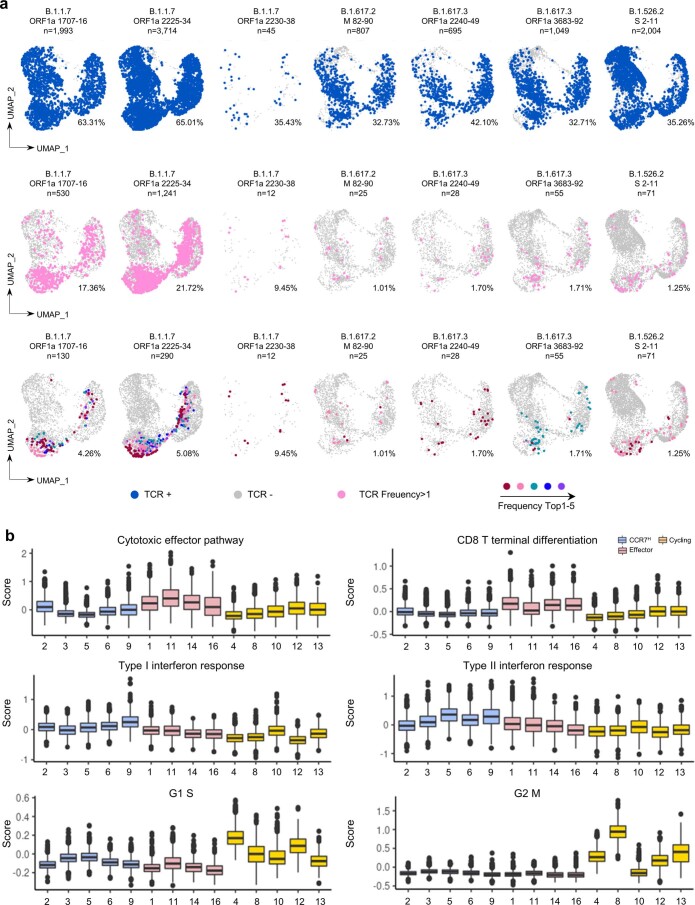
Single-cell TCR landscape and cell function scores of CD8 T cells specific to the top seven SARS-CoV-2 ancestral epitopes. (a) Same UMAP visualization as Fig. 7c, but with TCR sequence detection information (upper), TCR clonotype expansion (clonotype frequency > 1) information (middle), and top 5 most frequent TCR clonotype information projected on and split by each SARS-CoV-2 ancestral epitope. Source of selected ancestral epitopes, epitope IDs and total TCR number of TCRs with paired chains are labeled on the top of each panel. The percent of cells with TCR detected is shown in each panel. Except B.1.1.7 ORF1a 1707-16 and B.1.1.7 ORF1a 2225-34 epitope specific CD8 T cells, the highest TCR expansion frequency of others was less than 5. For B.1.1.7 ORF1a 2230-38 and B.1.617.3 ORF1a 2240-49 epitope specific CD8 T cells, the highest clonotype frequency was 2, therefore the corresponding cells are colored in dark red. Color-mapping are exclusive to each panel. (b) Boxplot of cell function scores for each CD8 T cell cluster. The cluster IDs on x-axis is the same as Fig. 7a. Number of cells (n) in each tested cluster are shown in Fig. 7a. The genes used in each gene panel for corresponding function score evaluation are listed in Table S6. The outlines of the boxes represent the first and third quartiles. The line inside each box represents the median, and boundaries of the whiskers are found within the 1.5×IQR value.

Based on the cell cytotoxicity assay, seven ancestral epitopes leading to substantial cytotoxic effects were finally selected as major SARS-CoV-2 epitopes of cellular immunity for detecting SARS-CoV-2 antigen-specific TCRs, as the inactivated SARS-CoV-2 vaccine was derived from the ancestral SARS-CoV-2 strain. To achieve this, we performed paralleled single-cell RNA sequencing (scRNA-seq) and single-cell TCR sequencing (scTCR-seq) for the seven selected ancestral epitope-specific CD8 T cells enriched by tetramer staining from newly recruited unvaccinated healthy donors (Methods). In total, we produced scRNA-seq and scTCR-seq from 21,900 CD8 T cells with TCR potentially specific to the seven SARS-CoV-2 epitopes. The CD8 T cells were grouped into 16 distinct clusters based on their transcriptome profiles, and the specific marker genes for each cluster were identified (Fig. 7a,b, Supplementary Table 5, Extended Data Fig. 6b,c and Methods). Based on the commonly expressed marker genes, the cell clusters were then grouped into four meta clusters: clusters 2, 3, 5, 6 and 9 were grouped and annotated as CCR7^high^ CD8 T; clusters 1, 11, 14 and 16 were annotated as effector CD8 T cells (*IFNG*, *KLRG1*, *NKG7*, *GZMB*, *GZMK* and *CD82*); clusters 4, 8, 10, 12 and 13 with high expression of genes related to cell proliferation and cell cycle (*H2AFZ*, *PCNA*, *CCNB1*, *MCM3*, *RRM2* and *HMGB2*) were named cycling CD8 T cells; clusters 7 and 15 with low expression of *CD3D* and *CD3G* were named CD3^low^ cells and considered as minor non-T cells (Fig. 7a,b, Supplementary Table 5 and Extended Data Fig. 6c). The exact TCR double-chain clonotype of nearly 47% of all analyzed cells was determined (Fig. 7c). When considering the CD8 T cell subtype and TCR clonotype together, it was evident that CD8 T cells with expanded clonotypes were mostly in effector and proliferating state (Fig. 7c, Extended Data Fig. 7a and Methods).

We then devised three gene panels related to T cell naivety, proliferation and cytotoxicity to characterize the SARS-CoV-2 epitope-specific CD8 T cells (Supplementary Table 6). The results indicated that (1) CCR7^high^ CD8 T cell clusters had relatively higher naivety score; (2) the cycling CD8 T cells were more proliferative; and (3) the effector CD8 T cell clusters were of higher cytotoxicity (Fig. 7d and Extended Data Fig. 7b). Furthermore, the assayed CD8 T cells with TCR clonotype that were more frequently detected (that is, higher clonotype expansion) tended to have lower cell naivety but higher cytotoxicity (Fig. 7e). This potentially implies that CD8 T cells with higher specificity to SARS-CoV-2 antigens were of higher cytotoxic effect^35^, and these analyzed T cells were of TCRs highly specific to the designated SARS-CoV-2 epitopes. Lastly, the high frequency of TCR clonotypes shared among clusters 1, 8, 11, 12, 13 and 14 potentially indicated the transition direction from cycling CD8 T cells to effector CD8 T cells specific to SARS-CoV-2 (Fig. 7f). This SARS-CoV-2 epitope-specific TCR clonotype information was critical for assessing T-cell-mediated immune response, which was next used in a TCR specificity machine learning framework to characterize the SARS-CoV-2 vaccine immune response of the young and old donors.

### Comparison of SARS-CoV-2-specific immune receptor repertoire

**Fig. 8:**
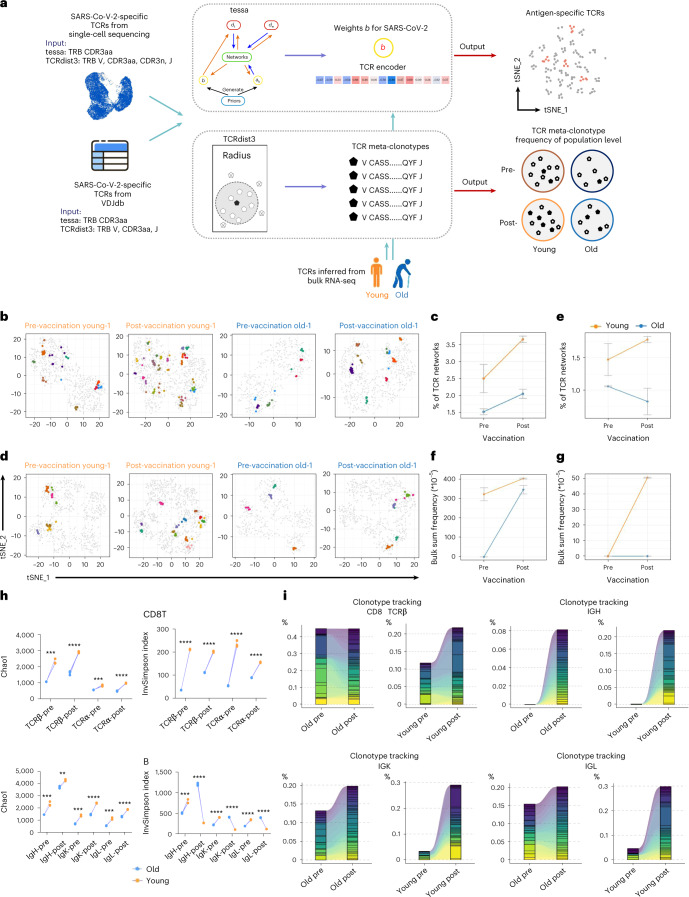
Comparison of TCR and BCR repertoires between young and old donors before and after vaccination. **a**, Schematic workflow for inferring SARS-CoV-2 antigen specificity for the CD8 T cells from young and old donors before and after vaccination using the tessa and TCRdist3 machine learning frameworks, respectively. CDR3aa, CDR3 amino acid sequence; CDR3n, CDR3 nucleic acid sequence. **b**, Exemplary t-distributed stochastic neighbor embedding (t-SNE) visualization of TCR clonotypes in the space of SARS-CoV-2 TCR embedding for representative young and old donors before and after vaccination. Networks containing ≥5 TCR clones are marked by colors. The same color in each panel represents that these TCRs are in the same network, and the color mapping is specific to each panel. **c**, Statistics of the proportion of TCR clonotype networks potentially specific to SARS-CoV-2 antigens before and after vaccination for young (*n* = 3) and old (*n* = 3) donors under the tessa machine learning framework. Data are shown as mean ± s.d. **d**, Same as **b** but for TCR clonotype visualization and projection using TCR embedding derived from literature reported S 269–277 YLQ SARS-CoV-2 epitope-specific TCRs and corresponding scRNA-seq data. **e**, Same as **c** but for TCR clonotype networks potentially specific to S 269–277 YLQ SARS-CoV-2 epitope. **f**, Statistics of sum of frequencies of TCRs within the radius of the centroid specific to SARS-CoV-2 antigens before and after vaccination for young (*n* = 3) and old (*n* = 3) donors under the TCRdist3 machine learning framework. Data are shown as mean ± s.d. **g**, Same as **f** but for the TCRdist3 model built with TCRs specific to S 269–277 YLQ SARS-CoV-2 epitope. **h**, Diversity estimation of vaccination-induced repertoire changes of CD8 T and B cells between the young and old donors. Higher value indicates greater diversity. *P < 0.05, **P < 0.01, ***P < 0.001 and NS, not significant. Wilcoxon rank-sum test (two-sided). **i**, Clonotype expansion of the top 40 CDR3 clonotypes for CD8 TCRβ, IgH, IgK and IgL for young and old groups before and after vaccination (7 days after the second dose).

**Extended Data Fig. 8:**
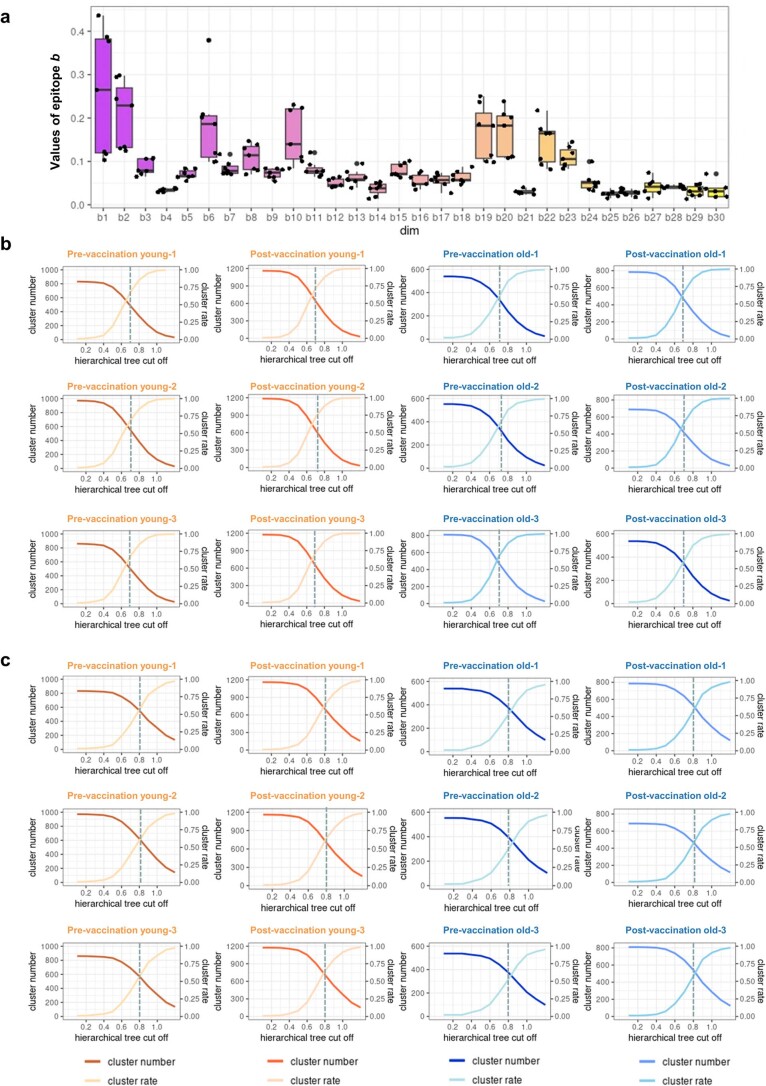
The weights of the TCR embedding derived from tessa, and the distribution of cluster numbers and cluster rate in hierarchical clustering for identifying SARS-CoV-2 specific TCR clone expansion. (a) The weights b of the SARS-CoV-2 specific TCR embeddings learned from tessa model. scRNA-Seq and scTCR-Seq data from SARS-CoV-2 epitope specific CD8 T cells (except for B.1.1.7 ORF1a 2230-38) as well as S 269-277 YLQ epitope specific CD8 T cells from literature were used in tessa for TCR embedding. The distribution of b across the 30 dimensions is highly similar to the TCR embedding weights derived from 19 antigen specific single-cell datasets in Zhang et al., which suggested the weights b learnt from the SARS-CoV-2 epitope specific single-cell data can be used for TCR embedding for SARS-CoV-2 epitope specific clonotype clustering. X-axis: the digits of the 30-dimensional embeddings. Y-axis: the weights value distribution of 7 SARS-CoV-2 epitopes calculated by tessa. The outlines of the boxes represent the first and third quartiles. The line inside each box represents the median, and boundaries of the whiskers are found within the 1.5×IQR value, each dot represent the actual value (n = 7). (b) The numbers of TCR clonotype networks and the clustering rates with different hierarchical clustering tree cutoffs in TCR clonotype data (after embedding by tessa) extracted from CD8 T bulk-cell RNA-Seq data (6 donors: 3 young and 3 old; pre- and post-vaccination). Cluster number (network number) and cluster rate gained a balance at the cutoff approximate to 0.7. Cluster rates were calculated as the number of TCR clonotypes that were in network size not equal to one divided by the total numbers of TCR clonotypes. Here, the TCR embeddings by tessa were produced from TCR clonotypes from the single-cell sequencing of CD8 T cells specific to SARS-CoV-2 epitopes identified in this study. (c) Same as Fig. S8b, but for tessa-based analysis with TCR clonotypes from the single-cell sequencing of CD8 T cells specific to SARS-CoV-2 S 269-277 YLQ epitope from literature. Cluster number (network number) and cluster rate gained a balance at the cutoff approximate to 0.8.

**Extended Data Fig. 9:**
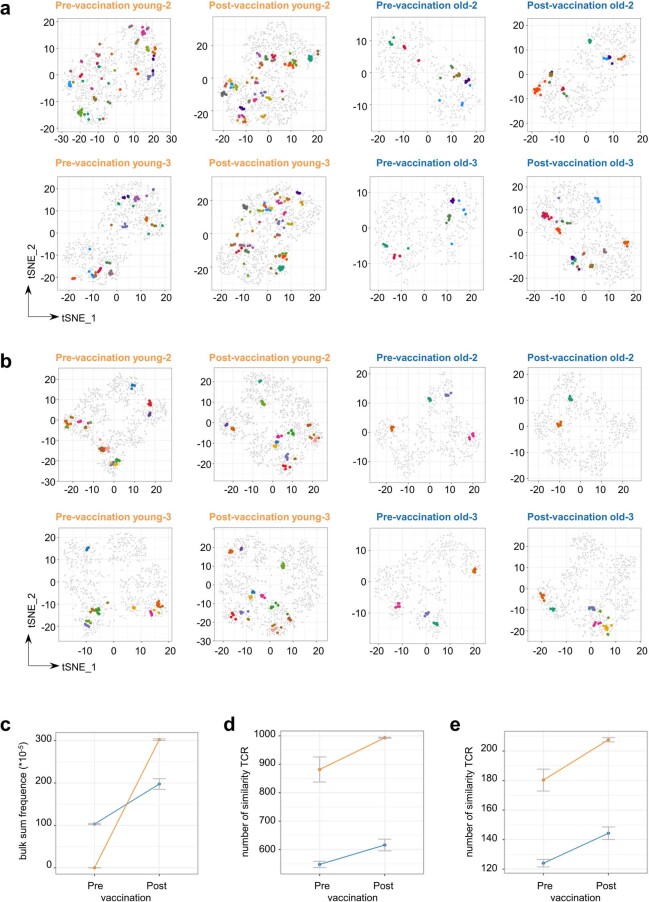
SARS-CoV-2 epitope TCR specificity prediction related results from tessa, TCRdist3 and FuzzyWuzzy analysis. (a) Additional tSNE visualization of TCR clonotypes in the space of SARS-CoV-2 TCR embedding for two representative young and old donors, before and after vaccination. TCRβ CDR3 sequences were derived from bulk-cell CD8 T cell RNA-Seq data from each donor. The embeddings were adjusted by the tessa-inferred weights specifically produced from TCR sequences of CD8 T cells specific to SARS-CoV-2 antigens shown in Fig. 5c and Fig. S7a (excluding B.1.1.7 ORF1a 2230-38 due to low number of captured cells and TCR sequences). Networks containing ≥5 TCR clones are marked by colors. These networks with high number of clonotypes clustered are deemed to be specific to SARS-CoV-2 antigens. The same color in each panel represents these TCRs are in the same network, and the color-mapping is specific to each panel. Networks are defined based on hierarchical clustering of TCRβ CDR3 embeddings modulated by tessa-inferred weights. (b) Same as a, but for TCR clonotype visualization and projection using TCR embedding derived from literature reported S 269-277 YLQ SARS-CoV-2 epitope specific TCRs and corresponding scRNA-Seq data. (c) Statistics of sum of frequencies of TCRs within the radius of the centroid specific to SARS-CoV-2 antigens pre- and post-vaccination, for young (n = 3) and old (n = 3) donors under TCRdist3 machine-learning framework. The meta clonotypes were discovered from TCRβ sequences specific to SARS-CoV-2 epitopes (excluding YLQPRTFLL) from VDJdb. Data are shown as mean ± SD. (d-e) Statistics of comparing the TCRβ repertoires of pre- and post-vaccination, young (n = 3) and old (n = 3) vaccinated individuals to the SARS-CoV-2 specific TCRβ sequences identified from our single-cell sequencing (d) and from VDJdb (e) by FuzzyWuzzy analysis. 90% CDR3 sequence similarity was considered as a match. Data are shown as mean ± SD.

The complexity and dynamics of immune cell receptor (that is, TCR and B cell receptor (BCR)) repertoire often reflect the capability and condition of host adaptive immune responses^36^. Therefore, we sought to characterize and compare the TCR and BCR repertoires of young and old donors before and after inactivated SARS-CoV-2 vaccine injection. We employed the tessa^37^ and TCRdist3 (ref. ^38^) machine learning algorithms to assess the SARS-CoV-2 epitope specificity of the CD8 TCR repertoires of the young and old donors before and after vaccination (Fig. 8a, Extended Data Figs. 8 and 9 and Methods). For tessa-based analysis, the SARS-CoV-2 antigen-specific TCR clonotypes (TCRβ CDR3 sequences only) identified from the CD8 T single-cell sequencing (Fig. 7c) were used to produce the epitope-specific weights for TCR embedding (Extended Data Fig. 8a). Then, the averaged weights across the SARS-CoV-2 antigens were applied to the TCRβ CDR3 sequences inferred from the bulk CD8 T cell RNA-seq data of each donor and vaccination condition for TCR embedding (Methods). Lastly, hierarchical clustering was performed on the embedding-transformed TCRs from each donor to identify networks of TCRs that are potentially specific to SARS-CoV-2 antigens (Fig. 8b, Extended Data Figs. 8b and 9a and Supplementary Table 7). It has been demonstrated that this workflow is informative for inferring antigen binding specificity^37^. Our results revealed that several TCR clusters with potentially high specificity to SARS-CoV-2 antigens were identified in the assayed donors before and after vaccination (Fig. 8b). Notably, the number of potential SARS-CoV-2 antigen-specific TCR clusters increased after vaccination across all individuals, reflecting TCR clonotype expansion induced by vaccine (Fig. 8b–g and Extended Data Fig. 9). In addition, there appeared to be more SARS-CoV-2 antigen-specific TCR clusters in the young donors both before and after vaccination compared to the old group (Fig. 8c), likely related to the better CD8 T-cell-mediated cytotoxic effect observed in the young group as described earlier (Figs. 4 and 5). We also performed the same tessa analysis procedures using scRNA-seq and TCR clonotype data specific to the previously reported S 269–277 YLQ SARS-CoV-2 epitope^39^, and the results revealed similar differences between young and old donors (Fig. 8d,e, Extended Data Figs. 8c and 9b and Methods). Additionally, independent validation analyses using TCRdist3 and a sequence matching method (FuzzyWuzzy; Methods) also suggested that young donors had a greater number of CD8 T cells specific to SARS-CoV-2 antigens with greater post-vaccination clonal expansion than old donors (Fig. 8f,g, Extended Data Fig. 9c–e and Methods).

**Extended Data Fig. 10:**
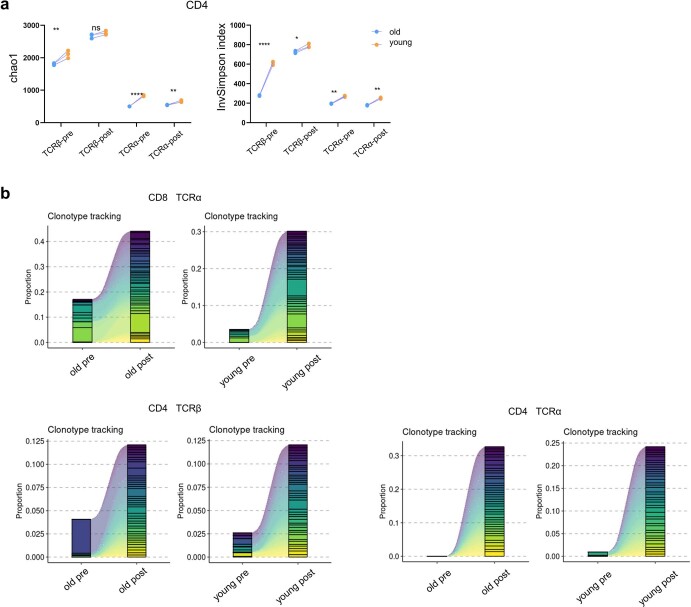
Comparison of CD4 and CD8 TCR repertoires between young and old donors before and after vaccination. (a) Diversity estimation of CD4 TCR repertoire in young (n = 3) and old (n = 3) donors before and after vaccination (7 days after the second dose). Higher value for greater diversity. *: *p* < 0.05; **: *p* < 0.01; ***: *p* < 0.001; ns: not significant. Wilcoxon Rank Sum test (two-sided). (b) Clonotype expansion of the top 40 CDR3 clonotypes for CD8 TCRα, CD4 TCRα, CD4 TCRβ for young and old groups before and after vaccination (7 days after the second dose).

We lastly used statistical methods to assess the complexity and diversity of TCR and BCR repertoires before and after vaccination for young and old groups. For the TCR sequences inferred from the RNA-seq data of CD8 T cells, both TCRα and TCRβ repertoires of the young group exhibited significantly higher complexity and diversity compared to the old group (Fig. 8h and Extended Data Fig. 10a). Similarly, the BCR repertoire of young individuals seemed to have higher complexity compared that of old individuals. In addition, higher degrees of clonotype expansion were observed for CD8 TCRβ, IgK and IgL from young individuals after vaccination (Fig. 8i and Extended Data Fig. 10b). Overall, these results indicated that the immune receptor repertoire of old donors was of lower richness and diversity than that of young donors, potentially explaining the weaker and slower neutralizing antibody production and cytotoxic effects after inactivated SARS-CoV-2 vaccine injection.

## Discussion

Aging is a critical risk for COVID-19 disease progression, severity and, especially, clinical outcome. Accordingly, it is important to dissect the altered immune response against SARS-CoV-2 in old individuals. Clinical test phase 2/3 results on mRNA and recombinant spike protein vaccine indicated relatively low antibody response and safety in individuals older than 60 years^18,40^. The mechanisms of impaired immune responses after vaccination in old individuals were obscure. The inactivated SARS-CoV-2 vaccines, CoronaVac and BBBIP, have also been demonstrated with high safety and efficacy for SARS-CoV-2 infection prevention^22^. Nevertheless, in the recent clinical trial, CoronaVac was shown to induce lower neutralizing antibodies in the old group than in the young group^22^. The inactivated SARS-CoV-2 vaccine was approved for the old population in July 2021 in China. Because of low incidence of COVID-19 in China, we were able to recruit enough old donors who were uninfected and unvaccinated for our study and to evaluate how inactivated SARS-CoV-2 vaccine-stimulated adaptive immune response was influenced by age. A comprehensive profiling of immune responses in the vaccinated population, especially in old individuals, would be greatly beneficial for optimization of vaccination regimens and development of better vaccines for SARS-CoV-2 variants.

T-cell-mediated immune response induced by different SARS-CoV-2 vaccines has been reported recently, but most of these studies relied only on IFNγ-based ELISpot assays. In the present study, we developed an epitope-based tetramer to detect antigen-specific CD8 T cells and sorted them for further analysis. To achieve this, we first identified the exact epitopes specific to SARS-CoV-2 virus with the combination of HLA-A2 binding assay and CD8 T cell activation and function assay. To be more coherent with the real world, we included epitopes with mutations introduced by the 13 major SARS-CoV-2 variant strains. We screened out 14 epitopes that induced strong CD8 T cell response but decreased response when mutated by a given variant. Most of them were in ORF1a, indicating the importance of ORF1a in T-cell-mediated immune response and potential immune escape after mutation. We also tested the previously reported HLA-A2-restricted SARS-CoV-2 epitope S 269–277 YLQ and its corresponding P272L mutant^30–33^. However, it requires more work to study the clinical relevance of these impaired immune responses caused by mutated epitope. Still, these epitopes raise concerns for further vaccine design in the future. In contrast, one and four epitopes were demonstrated with unchanged and increased CD8 T cell response after mutation, respectively, which also need further consideration for vaccine design.

T cell aging plays a pivotal role in predisposing older individuals to infections and in impairing responses to vaccinations^41^. It is known that the composition of T cells shifts toward sharply declined naive T cells and more developed memory T cells as age increases, leading to a reduction of the available TCR repertoire size^34,42^. These changes lead to a decline in the potential ability of the immune system in old individuals to resist new pathogens and a corresponding decrease in the defense against the outside world. The chronic inflammatory status, called ‘inflamm-ageing’, is the most important aspect of aging, which directly impacts on B lymphopoiesis and circulation. Besides, there is a decrease of naive B cells and an expansion of memory B cells in old individuals, and the ability of memory B cells to differentiate into plasma cells is impaired, resulting in an impaired ability to produce high-affinity protective antibodies when encountering new antigens^43^.

Low immune response in old individuals was observed in previous studies^44,45^, and our study demonstrated that the immune response induction in old individuals was not only weaker but also slower when compared to young individuals. Fifty days after the second dose, the neutralizing antibody titer in old individuals reached 57.5% of the young individuals at the same timepoint. For T cell response, antigen-specific CD8 T cells also increased from 0.35% to 1.02% in old individuals 50 days after the second dose. However, the antigen-specific CD8 T cells in old individuals reached only 12.68% and 30.81% of young individuals after 7 days and 50 days of the second dose, respectively. This indicated that CD8 T cell response was more difficult to be boosted than antibody response after inactivated SARS-CoV-2 vaccination in old individuals. This potentially implies that a third boost injection, or an optimized vaccination strategy, is required specifically for old individuals. A recent study demonstrated that a heterologous vaccination strategy of inactivated vaccine followed by mRNA booster elicited stronger immunity^46^.

The reasons for the mild symptoms experienced by the young adults have been demonstrated recently^21^. However, the mechanisms of slow and weak immune response in old adults were rarely explored in previous studies. In the present study, we attempted to address this issue from the perspectives of immune cell function and the amount of candidate cells ready to respond to vaccination. B cells, CD4 cells and CD8 T cells were sorted from the young and old pre-vaccinated and post-vaccinated donors for transcriptome analysis and BCR and TCR repertoire comparison. Accordingly, we provided a comprehensive experimental dataset and insights to address why the immune responses were weaker in old individuals after inactivated SARS-CoV-2 vaccine injection.

Comparison of the transcriptomes of B, CD4 and CD8 T cells from pre-vaccination and post-vaccination revealed genes potentially responsible for low immune responses in old individuals, functionally related to antigen processing and presentation. Under resting conditions before vaccination, immune cells from older individuals were enriched with upregulated genes associated with innate immune cell function, metabolic alterations and changes in antigen presentation on antigen-presenting B cells, suggesting potential ‘inflamm-ageing’ in old individuals^34^. After vaccination, immune cells in the younger group showed a strong upregulation of genes involved in immune responses. However, these enrichments were not found in the old donors but, rather, for pathways associated with cell migration, immunodeficiency and IgA production, suggesting potential defects in processing viral proteins from vaccines and presenting them as peptide antigens by lymphocytes in old individuals. In addition, the expression of genes that were implicated in coronavirus susceptibility was upregulated in a cell-subtype-specific manner with age. Notably, COVID-19 promoted age-induced immune cell polarization and gene expression related to inflammation^47,48^. Therefore, these findings suggest that immune system dysregulation and increased gene expression associated with susceptibility to SARS-CoV-2 may be at least partially accountable for poor vaccination effectiveness and vulnerability to COVID-19 in older adults.

Single-cell omics have been applied on SARS-CoV-2-related studies widely, most of them focused on diseases^49^. A recent study measured T and B cell repertoires after SARS-CoV-2 mRNA vaccine on memory lymphocytes but not antigen-specific cells^50^. We identified thousands of paired SARS-CoV-2 epitope-specific TCR sequences using scTCR-seq, which offers an important resource for further studying CD8 T cells specific to SARS-CoV-2. The TCRs reported here are limited to HLA-A2 MHC-I, and more work is needed for other HLA types.

In summary, we present a comprehensive analysis of adaptive immune responses before and after inactivated SARS-CoV-2 vaccine injection between young and old individuals. The old group exhibited slower and weaker (but still adequate) humoral immune response but markedly impaired cellular immune responses after vaccination compared to the young group. The underlying mechanisms were likely intrigued by altered immune cell function and decreased antigen-specific receptor repertoire diversity. Our work suggests that a third boost injection or an optimized vaccination strategy for older individuals needs to be urgently considered.

## Methods

### Human subjects enrollment

The institutional review board of the School of Medicine of Jinan University approved this study (JNUKY-2021-009). In total, 169 healthy volunteers were enrolled with written informed consent (Supplementary Table 1). The volunteers received the inactivated SARS-CoV-2 vaccine (CoronaVac or BBIBP-CorV) between June 2021 and October 2021. All volunteers were identified without a history or emergency infection of SARS-CoV-2 before and during the study with the questionnaire and viral test using PCR. Vaccinated donors were stratified in two major groups: the young group (18–30 years old, *n* = 121) and the old group (60–85 years old, *n* = 48). None of the participants experienced serious adverse effects after vaccination. Whole blood samples were collected at baseline (before vaccination), 14 days after the first vaccination dose and 7 days and 50 days after the second vaccination dose. The participants did not receive compensation.

### Isolation of plasma and peripheral blood mononuclear cells

Whole blood was collected in heparinized blood vacutainers and kept on gentle agitation until processing. Plasma samples were collected by centrifugation of whole blood at 600*g* for 10 minutes at room temperature without braking. The undiluted plasma was transferred to 1.5-ml cryotubes and stored at −80 °C for subsequent analysis. Peripheral blood mononuclear cells (PBMCs) were isolated by density gradient centrifugation using lymphocyte separation medium (GE Healthcare). Percentage of viability was estimated using standard trypan blue staining. The PBMCs were cryopreserved in FBS (LONSERA) with 10% DMSO (Sigma-Aldrich) and stored in liquid nitrogen until use.

### SARS-CoV-2-specific antibody measurements

Sandwich ELISA kits were used to detect SARS-CoV-2-specific antigen (S protein) neutralizing antibodies, IgG and IgM (2025-96, Leide Biosciences Co., Ltd.) in the collected plasma. In brief, SARS-CoV-2-specific antigens (S proteins) were coated on a 96-well plate. The collected samples and HRP-labeled second anti-human IgG antibody were added sequentially after washing of each step. The plate was added with TMB substrate and read on an iMark microplate reader (Bio-Rad) with the absorption at 450 nm and 630 nm. Optical density (OD) value = OD (450 nm) − OD (630 nm). The antibody titer was represented as the ratio of sample OD value (S) to the reference control OD value (CO)—that is, S / CO. The same operation was used to determine SARS-Cov-2-specific IgG and IgM levels.

### HLA-A2-restricted T cell epitope prediction

The spike (S), membrane (M), nucleocapsid (N), envelope (E) and ORF protein sequences of SARS-CoV-2 Wuhan-Hu-1 strain (NC_045512.2) were used for CD8 T cell epitope prediction with the MHC-I binding tool (http://tools.iedb.org/mhci). The prediction method used was IEDB Recommended 2.22 (NetMHCpan EL), with MHC allele selected as HLA-A*02:01, which is the most frequent class I HLA genotype among the Chinese population^28^. All predicted epitopes containing the same amino acid residue corresponding to the mutation from B.1.1.7 (Alpha), B.1.351 (Beta), P.1 (Gamma), P.2, P.3, B.1.429 (Epsilon), B.1.526.1 (Iota), B.1.526.2, B.1.618, B.1.617.1, B.1.617.2 (Delta), B.1.617.3 and B.1.1.529 (Omicron) were derived. The peptide with the best prediction score was used as the candidate epitope for the ancestral Wuhan-Hu-1 strain. Meanwhile, peptides with identical amino acid sequences except for the mutated points were used as candidate epitopes for variant B.1.1.7 (Alpha), B.1.351 (Beta), P.1 (Gamma), P.2, P.3, B.1.429 (Epsilon), B.1.526.1 (Iota), B.1.526.2, B.1.618, B.1.617.1, B.1.617.2 (Delta), B.1.617.3 and B.1.1.529 (Omicron). The above mutant strains were predicted to have a total of 239 mutant epitopes (relative to Wuhan-Hu-1). Epitopes from mutant strains with peptide length >12 aa and predicted antigen presentation ability <0.4 by VaxiJen 2.0 (http://www.ddg-pharmfac.net/vaxijen/VaxiJen/VaxiJen.html) were excluded, except for the B.1.1.7 and B.1.1.529 mutant strains (all ancestral and mutant peptides of these were synthesized). The previously reported immunodominant S 269–277 YLQ and the corresponding P272L mutant epitopes were also experimentally verified^30–33^. Finally, 122 ancestral and mutant epitope pairs were synthesized for downstream experiments (Supplementary Table 2).

### Peptide screening in T2 cells

The candidate peptides were synthesized by GenScript Biotechnology Co., Ltd. with purity >98% and resuspended in DMSO at a concentration of 10 mM. The titration of peptide concentration was performed as described previously^51^. The T2 cell line was shared by Anna Gil (University of Massachusetts Medical School). T2 cells are TAP-deficient T cells expressing HLA-A2 protein on the cell surface^52^. T2 cells were seeded into 96-well plates and then incubated with peptides at a final concentration of 20 µM at 37 °C for 4 hours. DMSO was set as blank control; the reported HLA-A2-restricted influenza A M1 peptide (M58-66 GILGFVFTL) was set as positive control; and the validated EBV virus peptide (IVTDFSVIK) was set as negative control^26,27,51^. Cells were stained with PE anti-human HLA-A2 antibody (BioLegend, 343305) at 4 °C in the dark for 30 minutes and acquired on a FACSCanto flow cytometer (BD Biosciences).

### HLA-A2 ELISA

Ninety-six-well U-bottomed plates were coated with 100 µl of 0.5 µg ml^−1^ streptavidin (BioLegend, 270302) at room temperature (18–25 °C) for 16–18 hours, washed three times with washing buffer (BioLegend, 421601) and blocked with dilution buffer (0.5 M Tris pH 8.0, 1 M NaCl, 1% BSA and 0.2% Tween 20) at room temperature for 30 minutes. Then, 20 µl of diluted peptide (400 µM) and 20 µl of conditional Flex-T monomer (200 µg ml^−1^) (BioLegend, 280003) were added into a 96-well U-bottom plate. To evaluate the outcome of UV-mediated HLA peptide exchange, a small aliquot of the exchange reaction mixture 300-fold in 1× dilution buffer was diluted and kept on ice until usage. DMSO was set as blank control; influenza A M1 peptide (M58-66 GILGFVFTL) was set as positive control; and EBV virus peptide (IVTDFSVIK) was set as negative control. Then, 100 µl of samples was added in duplicate and incubated for 1 hour at 37 °C. After washing three times with washing buffer, 100 µl of diluted HRP-conjugated antibodies (BioLegend, 280303) was added and incubated for 1 hour at 37 °C and then washed thoroughly. Next, 100 µl of substrate solution (10.34 ml of deionized water, 1.2 ml of 0.1 M citric acid monohydrate/tri-sodium citrate dihydrate pH 4.0, 240 µl of 40 mM ABTS and 120 µl of hydrogen peroxide solution) was added and incubated for 8 minutes at room temperature in the dark on a plate shaker at 300*g*. The reaction was stopped with 50 µl of stop solution (2% w/v oxalic acid dihydrate) and read at 414 nm in an ELISA reader within 30 minutes.

### Generation of antigen-specific HLA-A2 tetramer

Thirty microliters of peptide-exchanged monomer (produced in-house)^53^ was mixed with 3.3 µl of PE streptavidin (BioLegend, 405203) on a new plate and incubated on ice in the dark for 30 minutes. Then, 2.4 µl of blocking solution (1.6 µl of 50 mM biotin, Thermo Fisher Scientific, B20656) and 198.4 µl of PBS were added to stop the reaction and incubated at 4–8 °C overnight.

### Cell surface antibodies and tetramer staining

PBMCs were isolated from peripheral venous blood of healthy donors and SARS-CoV-2 vaccinees. The HLA-A2^+^ donors were identified by using flow cytometry without the subtype identification. In brief, 10^6^ PBMCs were stained with PE-conjugated anti-human HLA-A2 antibody (BioLegend, 343305) at 4 °C in the dark for 30 minuntes and acquired using a flow cytometer. HLA-A2^+^ PBMC samples were stimulated with T2 cells presenting SARS-CoV-2 epitopes for 16 hours and then stained with PE-labeled tetramer (produced in-house) plus APC-labeled human CD8 antibody (BioLegend, 344721). CD8 T cells isolated from vaccinated donors 50 days after second doses were co-cultivated with T2 cells loaded with SARS-CoV-2 epitopes (ORF1a 1707–1716, ORF1a 2225–2234, ORF1a 2230–2238, S 2–11, M 82–90, ORF1a 2340–2349 and ORF1a 3683–3692) at a 1:1 ratio, and PE-labeled tetramer with FITC-conjugated anti-human GZMB (BioLegend, 515403) were added after 7 days and acquired with a FACSCanto flow cytometer (BD Biosciences).

### Activation and cytotoxicity analysis of CD8 T cells

With the previously reported artificial antigen-presenting cell system from others and our studies, T2 cells expressing HLA-A2 were loaded with peptides for subsequent CD8 T cell activation. In brief, T2 cells were treated with 20 µg ml^−1^ mitomycin C for 30 minutes to stop cell proliferation^51^ and loaded with given epitope peptides for 4 hours. Peptide-loaded T2 cells were stained with FITC-conjugated anti-human HLA-A2 antibody (BioLegend, 343303) to analyze the loading rate. CD8 T cells were purified from PBMCs with EasySep Human negative selection (STEMCELL Technologies, 17953) with purity over 95%. Next, 0.25 × 10^6^ CD8 T cells isolated from healthy donors were co-cultured with 0.25 × 10^6^ peptide-loaded T2 cells stained with 5 µmol L^−1^ CFSE (TargetMol) and co-cultured with 1 µg ml^−1^ anti-human CD28 antibodies (BioLegend, 302901) and 50 IU ml^−1^ IL-2 (SL Pharmaceutical, recombinant human interleukin-2 (125Ala) injection). Then, 50 IU ml^−1^ IL-2 and 20 µM mixed peptides were supplemented every 2 days. The T cell activation markers CD69 (BioLegend, 310909) and CD137 (BioLegend, 309809) were evaluated after 16 hours, and tetramer-specific CD8 T cells and apoptosis marker Annexin V-APC (BioLegend, 640919) on T2 cells were evaluated after 7 days. On day 7, cells were re-stimulated with peptides for 4 hours in the presence of Leukocyte Activation Cocktail with GolgiPlug (BD Biosciences, 550583) plus 50 IU ml^−1^ IL-2, and the production of IFN-γ and GZMB was checked with PerCP-conjugated anti-human IFN-γ (BioLegend, 502524) and FITC-conjugated anti-human GZMB (BioLegend, 515403) staining.

### scRNA-seq experiment

Blood from unexposed donors was collected from healthy individuals registered at the Guangzhou Blood Center until July 2019. The donors had no known history of any systemic diseases, including, but not limited to, hepatitis B or C, HIV, diabetes, kidney or liver diseases, malignant tumors or autoimmune diseases. The samples were confirmed by negative reports of SARS-CoV-2 RNA real-time reverse transcription polymerase chain reaction (RT‒PCR) assays. PBMCs were isolated from seven randomly selected HLA-A2^+^ healthy donors using EasySep Human negative selection (STEMCELL Technologies, 17953) according to the manufacturerʼs instructions. Each type of epitope-specific CD8 T cell was generated with the method described above. Each epitope-specific CD8 T cell was labeled with PE-conjugated corresponding epitope-based tetramers and APC-conjugated anti-CD8 antibody and sorted by a FACSAria flow cytometer (BD Biosciences). Hashtags were used to label different epitope-specific CD8 T cells (Supplementary Table 2). Cell number and viability were checked after surface protein hashtag staining (cell viability >80%). Then, droplet encapsulation single-cell sequencing experiments were performed, and 10,000 living single cells were loaded onto each of the Chromium Controllers (10x Genomics). After droplet encapsulation, single-cell cDNA synthesis, amplification and sequencing libraries were generated using Chromium Single Cell 5′ Feature Barcode Library Kit (10x Genomics), Chromium Single Cell 5′ Library & Gel Bead Kit (10x Genomics) and Chromium Single Cell V(D)J Enrichment Kit (human T cell, 10x Genomics) according to the manufacturer’s instructions. The libraries from each loaded channel (up to eight channels) were multiplexed together and sequenced on an Illumina NovaSeq 6000.

### Single-cell sequencing data processing

10x Genomics Cell Ranger software (version 6.1.0) was used to process the FASTQ files with human reference GRCh38-2020-A (https://support.10xgenomics.com/single-cell-gene-expression/software/release-notes/build) for scRNA-seq and hashtag antibody sequencing, with default parameter settings. The resulting files were directly loaded into the R package Seurat (version 4.0.4) for further analysis. Cells with nFeature_RNA > 200 and nFeature_RNA < 6,000, as well as the percent of reads mapped to mitochondria genes <10%, were kept for FindVariableFeatures to extract the top 2,000 variable genes for subsequent analysis. The highest normalized hashtag count value was chosen to assign each cell to the corresponding epitope-specific sample, except for S 2–11 (sequenced without mixture). FindClusters (resolution = 1) was used to divide the cells into 16 clusters with the first ten principal components chosen from PCA analysis. The top ten marker genes for each cluster were identified by FindAllMarkers (Supplementary Table 5). AddModuleScore was used to calculate the score for the assigned gene set (Supplementary Table 6), and repOverlap from the R package immunarch (version 0.6.6) was used to aggregate the shared TCR clonotypes between different clusters^54^.

The scTCR-seq raw FASTQ files were aligned to human reference GRCh38 (version 3.1.0) (https://support.10xgenomics.com/single-cell-vdj/software/release-notes/3-1) with default parameters. Only TCR clonotypes with paired TRAV-CDR3-TRAJ and TRBV-CDR3-TRBJ-TRBC chains were conjoined to scRNA-seq data (Supplementary Table 8).

### RNA extraction and sequencing

CD8 T, CD4 T and B cells were purified from PBMCs with EasySep Human positive or negative selection (STEMCELL Technologies, 17953, 17852 and 17954) according to the manufacturerʼs instructions. The cell purities were checked for over 95% with anti-human CD8 (BioLegend, 344721), anti-human CD4 (BioLegend, 317408), anti-human CD19 (BioLegend, 392504) and anti-human CD20 (BioLegend, 302326), respectively. Total RNA was isolated from CD8 T, CD4 T and B cells of three randomly selected young and old donors individually at baseline (before vaccination) and 7 days after the second vaccination dose by using TRIzol reagent (Invitrogen) (Supplementary Table 1). RNA purity was checked by the NanoPhotomerer spectrophotometer (IMPLEN), and integrity was assessed using the RNA Nano 6000 Assay Kit of the Bioanalyzer 2100 system (Agilent Technologies). Then, cDNA libraries were constructed using 0.1 µg of RNA per sample with the NEBNext UltraTM RNA Library Prep Kit for Illumina (New England Biolabs) following the manufacturer’s recommendations; the libraries were sequenced on an Illumina NovaSeq platform; and 250-bp paired-end reads were generated.

### RNA-seq data analysis

FASTQ files from CD4 T, CD8 T and B cells of the young and old donors before and after vaccination were aligned to human reference genome Homo_sapiens_Ensemble_94 by HISAT2 (version 2.0.5) after quality trimming. FeatureCounts was used to generate a raw gene expression count for each sample. The R package DESeq2 (version 1.32.0) was applied to perform differential expression analysis. DEGs were identified with adjusted *P* < 0.05 and absolute log_2_ fold changes > 1 (Supplementary Table 3). KEGG pathway enrichment analysis for DEGs was performed with the R package topGO (version 2.44.0). KEGG pathways with *P* < 0.05 were considered significantly enriched (Supplementary Table 4). The normalized expression matrix from DESeq2 was further centered and scaled by scale function and then visualized by the R package pheatmap (version 1.0.12).

### Bulk cell BCR and TCR analysis

The bulk cell BCR and TCR repertoire were extracted from the CD4 T, CD8 T and B cell RNA-seq data generated in this study by using MixCR (version 3.0.13)^55^ (https://github.com/milaboratory/mixcr), with default parameter settings. Each TCR repertoire contains the information of TRAV-CDR3-TRAJ and TRBV-CDR3-TRBJ-TRBC, and each BCR repertoire contains the information of V, D and J regions of IgH, IgK and IgL. The changes in the abundance and diversity of the TCR and BCR repertoire in young and old before and after vaccination were characterized with chao1 and inverse Simpson index as described previously^34^ and calculated using the R package immunarch (version 0.6.6)^54^. A *t*-test was used to characterize the significance of difference in chao1 and inverse Simpson between the young and old groups.

### Prediction of antigen-specific TCRs

tessa^37^ (https://github.com/jcao89757/tessa) is a model to quantitatively interpret the functional relevance of T cell repertoire that identifies TCR clonotypes in the same network having similar functions and may be specific to the same antigen. The input files of tessa are scRNA-seq expression and scTCR-seq CDR3β data matched through cell barcode. Weight *b* is an important parameter for TCR embeddings. Similar TCRs defined by the weighted embeddings are grouped into TCR networks reflective of antigen specificity. We calculated weight *b* for the SARS-CoV-2 antigen-specific scRNA-seq and scTCR-seq data described above to gain the *b* value for each epitope. At the same time, we encoded bulk-cell CDR3β sequences extracted from CD8 T bulk-cell RNA-seq to form a 30-dimensional numerical vector (TCR embeddings) and gave each CDR3β a new 30-dimensional tessa-inferred TCR embedding through multiply by the average of *b* learned from seven epitopes (ORF1a 2230–2238 not used due to low number of captured specific cells and TCR sequences) specific CD8 T single-cell sequencing datasets. For each bulk-cell TCR repertoire, we performed hierarchical clustering by hclust function under Manhattan distance after TCR embedding. In addition, the cutree function with varying the parameter *h* from 0.0 to 1.5 was used to calculate cluster numbers and cluster rate to find the best cutoff value. These networks with a high number (≥5) of clonotypes clustered together are deemed to be specific to SARS-CoV-2 antigens.

TCRdist3 (ref. ^38^) (https://github.com/kmayerb/tcrdist3) is a distance-based TCR repertoire analysis algorithm. TCRdist3 was used to further validate the change between young and old individuals, before and after vaccination, using bulk-cell sequencing TCRs. The input files of TCRdist3 are TCRβ V, CDR3 and J regions of seven selected epitope-specific TCRs through above single-cell sequencing. Meta-clonotype discovery pipeline was used to find the meta-clonotype specific to the seven selected epitopes. All meta-clonotype files (theta = 1 × 10^5^) and each bulk-cell TCR repertoire were used as the input files to the meta-clonotype tabulation pipeline. The sum of TCR frequencies within the radius of centroid was counted for each bulk-cell TCR repertoire respectively as the final result.

FuzzyWuzzy (https://github.com/seatgeek/fuzzywuzzy), a string-matching algorithm, was used to compare the TCR repertoires of vaccinated individuals to the SARS-CoV-2-specific TCR sequences. Ninety percent similarity was used as a sequence match threshold.

### VDJdb data analysis

VDJdb (https://vdjdb.cdr3.net/) is a curated database of TCR sequences with known antigen specificities. We extracted SARS-CoV-2 epitope-specific TCRs with the filter parameters as ‘HomoSapiens’ ‘HLA-A*02’ ‘HLA-A*02:01’ and ‘HLA-A*02:01:48’. After filtering, 4,125 TCRs, including TCRα and TCRβ, were obtained. These TCRs were used to generate weight *b* from the tessa machine learning framework and to produce meta-clonotypes for the TCRdist3 machine learning framework as well as comparison with TCRs from bulk-cell sequencing of the vaccinated cohort and the TCRs specific to the seven selected epitopes (Supplementary Tables 9 and 10).

### Statistics and reproducibility

The difference in adaptive immune response, which includes antibody response and cellular immune response between young and old donors before the first dose of inactivated SARS-CoV-2 vaccine, 14 days after the first dose and 7 days and 50 days after the second dose, were analyzed by one-way ANOVA and paired-sample *t*-tests (two-sided). Significance was achieved when *P* < 0.05 where appropriate. Data distribution was assumed to be normal, but this was not formally tested. No data points were excluded for analysis. No statistical methods were used to pre-determine sample size, but our sample sizes are similar to those reported in previous publications^56,57^. All data collection and statistics were performed in GraphPad Prism 8, SoftMax Pro 7.1.1 GxP, SPSS 22.0, FlowJo (10.7) and the R statistical package. Samples were allocated to corresponding age group (young: <60 years; old: ≥60 years) without randomization. All the sample information was blinded during all the experiments. Samples were unblinded only for data analysis and cross-group comparison.

### Reporting summary

Further information on research design is available in the Nature Portfolio Reporting Summary linked to this article.

## Supplementary information

Reporting Summary

Supplementary Tables 1–10

## Data Availability

The sequencing data reported in this paper were deposited in the Gene Expression Omnibus with accessions numbers GSE191088 and GSE191089. TCRs from published publications were downloaded from https://vdjdb.cdr3.net/. Human reference GRCh38-2020-A was downloaded from https://support.10xgenomics.com/single-cell-gene-expression/software/release-notes/build. Human reference GRCh38 (version 3.1.0) was downloaded from https://support.10xgenomics.com/single-cell-vdj/software/release-notes/3-1.

## Source data

Source Data Extended Data Fig./Table 1 Statistical source data of flow cytometry.

## Code availability

The R packages and other analytical code used in this study are all from open sources and are available from websites described in Methods.
